# Boninite-like intraplate magmas from Manihiki Plateau require ultra-depleted and enriched source components

**DOI:** 10.1038/ncomms14322

**Published:** 2017-02-09

**Authors:** Roman Golowin, Maxim Portnyagin, Kaj Hoernle, Folkmar Hauff, Andrey Gurenko, Dieter Garbe-Schönberg, Reinhard Werner, Simon Turner

**Affiliations:** 1 GEOMAR Helmholtz Centre for Ocean Research Kiel, Wischhofstrasse 1-3, 24148 Kiel, Germany; 2 V.I.Vernadsky Institute of Geochemistry and Analytical Chemistry, Kosigin street 19, Moscow 119991, Russia; 3 Christian-Albrechts-University of Kiel, Ludewig-Meyn-Strasse 10, 24118 Kiel, Germany; 4 Centre de Recherches Pétrographiques et Géochimiques, UMR 7358, Université de Lorraine, 54501 Vandoeuvre-lès-Nancy, France; 5 Department of Earth and Planetary Sciences, Macquarie University, New South Wales 2109, Australia

## Abstract

The Ontong Java and Manihiki oceanic plateaus are believed to have formed through high-degree melting of a mantle plume head. Boninite-like, low-Ti basement rocks at Manihiki, however, imply a more complex magma genesis compared with Ontong Java basement lavas that can be generated by ∼30% melting of a primitive mantle source. Here we show that the trace element and isotope compositions of low-Ti Manihiki rocks can best be explained by re-melting of an ultra-depleted source (possibly a common mantle component in the Ontong Java and Manihiki plume sources) re-enriched by ≤1% of an ocean-island-basalt-like melt component. Unlike boninites formed via hydrous flux melting of refractory mantle at subduction zones, these boninite-like intraplate rocks formed through adiabatic decompression melting of refractory plume material that has been metasomatized by ocean-island-basalt-like melts. Our results suggest that caution is required before assuming all Archaean boninites were formed in association with subduction processes.

## Abstract (web summary)

Large igneous provinces may form very quickly, but compositions often differ. Here, the authors find that boninite-like rocks at Manihiki are a result of re-melting of an ultra-depleted source therefore putting into question that all boninitic rocks on Earth must be related to subduction processes.

Large igneous provinces are formed over a relatively short time period (several million years) through massive volcanism related to pressure-release melting of upwelling mantle plume heads^1^,^2^,^3^. Some basement lavas from the Ontong Java (OJP), Manihiki and Hikurangi Plateaus, located in the Southwest Pacific Ocean (Fig. 1), have similar chemical compositions and ages (∼118–125 Myr), consistent with their derivation from a common source through high degrees (∼30%) of mantle melting^4^,^5^,^6^,^7^. It has been proposed that they represent fragments of a single ‘super' plateau^8^,^9^ and that this magmatic event, the largest in the Phanerozoic, covered ∼1% of Earth's surface^5^.

The primitive Kroenke and more evolved but isotopically identical and dominant Kwaimbaita basement lavas from OJP^6^,^10^,^11^ are thought to be derived from a primitive, ancient, lower-mantle reservoir^12^. These lavas have flat, primitive-mantle-normalized incompatible-element patterns. The stratigraphically younger and minor Singgalo lavas^10^,^11^ at OJP have geochemical characteristics similar to Manihiki high-Ti basement lavas^4^, for example, drilled at DSDP Site 317 (ref. 5). Owing to their possibly younger stratigraphic age, we do not consider these lavas further in this paper.

In contrast to OJP, a major part of the Manihiki Plateau is composed of relatively low-Ti, high-MgO rocks, dated at 118–125 Myr (refs 4, 13), and related to the basement (main) phase of plateau formation similar to OJP Kroenke/Kwaimbaita lavas. These low-Ti rocks have variable contents of the most incompatible elements, positive relative Nb-Ta anomalies, and most of them have U-shaped patterns on primitive mantle- and mid-ocean-ridge basalt (MORB-)-normalized incompatible element diagrams^4^,^13^. The boninite-like, U-shaped patterns are unique and enigmatic for plume-derived intraplate magmas, indicating depletion and re-enrichment processes involved in their genesis. In previous studies, the origin of the low-Ti rocks was explained by high-degree melting of a mixed source containing depleted mantle wedge material and small amounts of subducted volcaniclastic sediments^13^ and by extensive melting of a hybrid mantle source comprising FOZO-type peridotite and recycled oceanic crust^4^.

Here we present geochemical data from rare, fresh glass samples and additional major element data from bulk rock samples from the Manihiki Plateau obtained by dredging during the R/V Sonne SO193 (ref. 4) and SO225 expeditions (Fig. 1). Glass samples from the SO193 cruise yielded ages of 122.9±1.6 and 124.5±1.5 Myr (ref. 4). The fresh glasses provide a unique opportunity for precise geochemical characterization of the parental Manihiki melts, not possible with phenocryst-bearing whole rock samples variably affected by seawater alteration. We show that the origin of the low-Ti Manihiki rocks is related to re-melting of an isotopically distinctive, highly depleted plume source beneath Manihiki and possibly the OJP, comprising small and variable amounts of a recycled HIMU (high time-integrated ^238^U/^204^Pb ratio)-like component.

## Results

### Composition of low-Ti glasses

Relics of fresh glasses were found in strongly palagonitized hyaloclastites and pillow-lava margins. The glasses contain very rare vesicles and fresh olivine micro-phenocrysts with inclusions of chromium-rich spinel. The glasses are tholeiitic (SiO_2_=51.1–52.4 wt%; Na_2_O+K_2_O=1.5–1.8 wt%), characterized by very low TiO_2_ (0.27–0.44 wt%) and relatively high MgO (9.0–9.8 wt%) contents (Fig. 2a,b, Supplementary Table 1). Some glass and whole-rock samples have SiO_2_ contents above 52 and as high as 56 wt% at MgO> 8wt%, which is characteristic for boninites^14^. High CaO contents (12.9–13.2 wt%) reflect insignificant clinopyroxene fractionation consistent with its absence as a phenocryst phase. The low-Ti glasses also have low but variable K_2_O contents (0.02–0.17 wt%) at relatively constant and high Mg# (molar Mg/(Mg+Fe)=0.65–0.67). Compared with primitive Kroenke lavas from the OJP basement with similar MgO content (9–10 wt% MgO; ref. 15), the low-Ti Manihiki glasses have higher SiO_2_ (51.4–52.4 versus ∼48.9–50.4 wt%) and lower TiO_2_ (0.27–0.44 versus ∼0.68–0.78 wt%) contents. The low-Ti Manihiki glasses contain 0.13–0.25 wt% H_2_O, 350–546 p.p.m. S and 126–242 p.p.m. Cl (Supplementary Table 1 and ref. 4), which are similar to those in quenched glasses and olivine-hosted melt inclusions from OJP^15^,^16^. The most K_2_O-rich glasses (0.09–0.18 wt% K_2_O) have Cl/K_2_O=0.10–0.14, H_2_O/K_2_O=1.4–1.5 and H_2_O/Ce∼200, which are within the range of typical MORB and OIB melts^17^,^18^,^19^. The K_2_O-poor glasses (∼0.02 wt% K_2_O) have high Cl/K_2_O=0.61–0.71, H_2_O/K_2_O=6.3–7.0 and H_2_O/Ce=1,191–1,258. The relative enrichment of the glasses in Cl and H_2_O may indicate magma assimilation of a sea-water-derived component in the crust^16^,^19^,^20^. However, Cl and H_2_O in the Manihiki glasses exhibit strong positive correlations with incompatible trace elements, whose concentrations are very low in seawater (for example, Th, Nb, rare earth elements (REE)). Such correlations preclude significant H_2_O and Cl degassing from magmas before and during eruption and are unexpected if magmas assimilated variable amounts of seawater-derived components, typically brine^21^. Instead, the relative Cl and H_2_O enrichment in low-Ti glasses may reflect their source composition, which might be similar to the incompatible-element depleted sources of komatiites from the Abitibi Belt, Canada and Gorgona Island, eastern Pacific^22^,^23^.

The low-Ti Manihiki glass samples exhibit variable contents of highly incompatible elements and display U-shaped patterns on incompatible-element diagrams with concentrations normalized to N-MORB (Fig. 2c). U-shaped patterns are also particularly well expressed by the REE normalized to chondrites (Fig. 2d). The U-shaped patterns in Manihiki glasses are similar to those in high-Ca boninites from the Troodos ophiolite on Cyprus and the Tonga Arc, whereas Kroenke-type OJP rocks have relatively flat trace element patterns (Fig. 2c). The low-Ti Manihiki glasses are more depleted in moderately incompatible elements (Lu to Zr) than primitive Kroenke lavas, whereas more incompatible elements (for example, light rare earth elements (LREE), Nb, Ta, Th, U, Ba, Rb) in low-Ti Manihiki glasses range from being more depleted to more enriched (Fig. 2c). Both, low-Ti Manihiki and Kroenke/Kwaimbaita lavas show strong relative depletion in K relative to LREE and relative enrichment in Nb-Ta in the normalized incompatible-element patterns that distinguishes them from subduction-zone-related boninites from Troodos and Tonga, as does the lack of strong relative Pb enrichment of the low-Ti Manihiki glasses (Fig. 2c).

Isotope compositions of the low-Ti glass samples fall into two clusters forming positive correlations on the ^87^Sr/^86^Sr versus ^143^Nd/^144^Nd and the Pb isotope diagrams, and negative correlations on ^206^Pb/^204^Pb versus ^143^Nd/^144^Nd and ^87^Sr/^86^Sr isotope diagrams (Fig. 3, Supplementary Fig. 1). The most incompatible-element-depleted low-Ti tholeiites have isotopic compositions similar to enriched mid-ocean-ridge basalts (E-MORB; Fig. 3, Supplementary Fig. 1). Glasses with the strongest enrichment in the most incompatible elements have the most radiogenic Pb (Supplementary Table 1) and the least radiogenic Sr and Nd isotope signatures, and trend towards the compositions of some HIMU-like ocean-island basalts (OIBs).

## Discussion

Manihiki low-Ti tholeiites share a number of characteristics with boninites from subduction-related settings, which are defined as rocks with >52 wt% SiO_2_, >8 wt% MgO and <0.5 wt% TiO_2_ (ref. 14). Two glass samples, as well as a significant number of whole rock samples, fulfil the major element criteria for boninites in terms of SiO_2_, TiO_2_ and MgO content (Fig. 2a,b, Supplementary Table 1, 2). In addition, both the low-Ti Manihiki rocks and boninites have U-shaped incompatible element patterns with low Sm/Yb but high La/Sm (Fig. 2c,d), and low Ti/V (<12) ratios. Based on high CaO/Al_2_O_3_ ratios (0.83–0.88), the mafic low-Ti Manihiki tholeiites are similar to high-Ca-boninites^24^, such as, for example, those from the Tonga Arc^25^ and the Troodos upper pillow lavas on Cyprus^26^, indicating their origin from a clinopyroxene-bearing, depleted lherzolite or harzburgite source^27^.

A major process for generating the characteristic compositional features of boninites is melting of highly depleted (through previous melt extraction) peridotitic mantle, triggered through addition of a hydrous slab-derived fluid or melt, enriched in incompatible elements^25^. The hydrous fluid/melt lowers the solidus temperature of the depleted peridotite, causing it to melt^24^,^28^. Several geochemical characteristics of the low-Ti Manihiki magmas, however, are not consistent with a subduction origin. There is no evidence of strong, selective enrichment in highly fluid-mobile elements (for example, Rb, Sr, U, Pb). Further, the strong enrichment in Nb and Ta compared with other incompatible elements is unusual for subduction-related magmas (Fig. 2c). In addition, the Manihiki glasses have low H_2_O contents (0.13-0.25 wt%, see Supplementary Table 1 and ref. 4). Finally, palaeo-reconstruction studies indicate that the plateau basement formed in an oceanic intraplate setting, possibly near a mid-ocean-ridge spreading centre, but far away from a subduction zone^8^,^29^.

In contrast to the enriched fluid or melt component in boninites, which is derived from the subducting slab, we show in the following discussion that the re-enrichment of the ultra-depleted mantle source at Manihiki most likely occurred through addition of an enriched HIMU-like mantle plume component to the source. Unlike hydrous fluids involved in boninite petrogenesis, the small amounts (≤1%, see below) of this HIMU mantle component probably did not have a major affect on the degree of mantle melting, and instead were mainly responsible for the variability in the concentrations of highly incompatible elements and Sr-Nd-Pb isotope ratios in low-Ti Manihiki magmas.

Variations of radiogenic isotope ratios (Fig. 3; Supplementary Fig. 1; Supplementary Table 1) in the low-Ti Manihiki glasses indicate that they do not simply represent different melt fractions from a single homogeneous mantle source. Following the criteria of ref. 30, linear correlations between incompatible element and isotope ratios with the same denominator (Supplementary Fig. 2) allow us to narrow down the range of possible scenarios for the origin of Manihiki magmas, indicating either mixing of two magmas from different sources (‘magma mixing') or fixed degree of melting of a heterogeneous mantle source, comprising different proportions of depleted and enriched components (‘source mixing'), for example, metasomatism of a depleted source with variable amounts of an enriched component. Although these two petrogenetic scenarios cannot be distinguished solely on the basis of incompatible-element (Supplementary Fig. 3) and isotope systematics^30^, they imply different mass fractions of the two components and consequently different effects on the major element composition of the magmas.

Before we can determine the compositions of the depleted and enriched end members, we need to calculate the compositions of primary Manihiki magmas from the composition of the low-Ti glasses. We calculated the primary magma compositions using the PRIMELT2 model^31^. The glasses are saturated with olivine and spinel, which are the only liquidus phases, and thus the application of the model, involving incremental addition of olivine to the evolved melt, is straightforward. The calculated primary magmas are in equilibrium with olivine Fo_91.1–91.3_, have high MgO (15.7–17.0 wt%) and CaO (10.4–11.0 wt%), and are peridotite-derived compositions (Supplementary Table 3). Concentrations of incompatible elements in the primary magmas were then calculated by dilution of the trace element concentrations in glasses proportional to the amount of olivine added to the glass compositions.

To estimate the composition of the depleted source component, we first carried out forward modelling of pooled fractional melting, using a primitive mantle composition^32^ and bulk partition coefficients for peridotite melting^33^ to derive melts with similar concentrations of moderately incompatible elements, for example, heavy rare earth elements (HREE), to those in the calculated low-Ti primary melts (Fig. 4, Supplementary Fig. 4). The results of the forward modelling show that up to 15% fractional melting of fertile or moderately depleted mantle peridotite cannot explain the strong depletion of the Manihiki melts in moderately incompatible elements (Fig. 4a). The required depletion can only be achieved with multi-stage melting, when a significantly depleted source (through previous melting) is re-melted, and the magmas produced during the second stage of melting do not mix with magmas of the initial stage of melting, implying a time gap between these two melting events. The best-fit parameters of the two-stage melting scenario obtained by the least-squares fitting of the HREE concentrations in the modelled melts to the Manihiki primary magmas are ∼11% melting of fertile peridotite during the first stage and ∼9% melting of the residual mantle during the second stage. Modelling with more depleted sources than primitive mantle (for example, Depleted MORB Mantle (DMM) type source^33^) results in decreasing the estimate for the total extent of melting. Taking into account the uncertainties in the physical parameters of melting and the initial source composition, the estimates in Fig. 4a only provide a possible (but not unique) origin for the depleted end member of the low-Ti Manihiki primary melts. Although the degrees of melting of the individual melting stages cannot be fully constrained, the conclusion about the necessity of two (or multi)-stage melting to explain the concentrations of moderately incompatible elements in primary Manihiki magmas is robust. In conclusion, the modelling results show that the depleted end member involved in the origin of the low-Ti Manihiki magmas could be highly depleted peridotite (that is, ultra-depleted mantle, UDM; Supplementary table 4) residual after ∼11% fractional melting and melt removal.

Re-melting of residual mantle peridotite (and subsequent re-enrichment in incompatible elements) points to a similar type of origin for the low-Ti intraplate rocks and subduction-related boninites^24^. Since the low-Ti rocks do not show any evidence for a subduction-related origin as discussed above, elevated temperature and further decompression melting related to the emplacement of a hot mantle plume head are likely to have driven the melting under the Manihiki Plateau rather than a hydrous fluid/melt. The overall lower SiO_2_ content of the low-Ti tholeiites compared with boninites can also be explained through dry refractory lherzolite melting, since hydrous melting of refractory lherzolite would increase the contribution of orthopyroxene to the melt resulting in a higher SiO_2_ melt content^28^.

A recent study described ultra-depleted melt inclusions in olivines from a Kroenke-type lava (Fig. 2a,b,d), which were proposed to originate from a previously unrecognized ultra-depleted component in the OJP mantle source^16^. Since OJP, Hikurangi and Manihiki Plateaus are likely to have formed as a single plateau originally^8^,^9^, they presumably shared some similar source material. The recognition of UDM in the source of the low-Ti Manihiki magmas suggests that this mantle component may be involved in the origin of both OJP and Manihiki magmas.

The isotopic composition of the UDM end member can place important constraints on its origin. Since the modelled UDM incompatible element pattern shows systematically decreasing abundances of elements as they become more incompatible with the most incompatible elements (Rb through Ta) showing near zero abundances (<0.001; Fig. 4a, Supplementary Table 4), the most-depleted samples cannot serve as the depleted end member, due to their U-shaped patterns. Following the approach of ref. 30, the isotope composition of the UDM was estimated from linear correlations of the Manihiki low-Ti lavas on plots of x/Pb versus Pb isotope ratios, x/Nd versus ^143^Nd/^144^Nd and x/Sr versus ^87^Sr/^86^Sr (where x is a highly incompatible element, for example, Ba, which has a negligible—near zero—concentration in the residual mantle after fractional melting). Since the Ba concentration in the UDM is near to zero, ratios of Ba/Pb, Ba/Nd and Ba/Sr will also approach zero and thus the UDM will have the following isotopic composition: ^143^Nd/^144^Nd=0.51285±0.00001, ^206^Pb/^204^Pb=18.00±0.16, ^207^Pb/^204^Pb=15.48±0.03, ^208^Pb/^204^Pb=38.07±0.11, ^87^Sr/^86^Sr=0.70373±0.00007 (Supplementary Fig. 2, Supplementary Table 5; see also Fig. 3, Supplementary Fig. 1). Higher ^208^Pb/^204^Pb and more radiogenic ^87^Sr/^86^Sr compared with Pacific MORB (Fig. 3, Supplementary Fig. 1) are not consistent with an origin of the UDM end member through melting of depleted Pacific MORB mantle in the lithosphere or asthenosphere above the plume at the time of formation of the Ontong Java and Manihiki plateaus. Furthermore, the more radiogenic ^143^Nd/^144^Nd and less radiogenic Pb isotope ratios exclude any connection of the UDM to the presumably dominant Kroenke/Kwaimbaita lavas at OJP. The very high ^147^Sm/^144^Nd parent/daughter ratio of ∼0.57 in the UDM end member (assuming 10% previous melt extraction from its source, see Supplementary Table 4) places strong constraints on the timing between the depletion and re-enrichment (metasomatic) events. If the interval was ≥18 Myr, the *ɛ*Nd(t) for the UDM would have been <0. Therefore, the time interval between the events is likely to have been within a few million years unless the original UDM source was isotopically very enriched having very unradiogenic Nd isotopic composition (reflecting very low time-integrated ^147^Sm/^144^Nd). Even the ^147^Sm/^144^Nd of the two depleted samples (derived from the re-enriched UDM source) is high (up to 0.324) and thus the depletion event of the UDM source is unlikely to have taken place more than several hundred million years ago or the original UDM source was very enriched (*ɛ*Nd(t)<0 if more than 400 Myr ago). The elevated ^208^Pb/^204^Pb (at its ^206^Pb/^204^Pb, plotting above the Pacific MORB field) and relatively radiogenic ^87^Sr/^86^Sr isotopic composition of the UDM end member overlaps almost completely with the range of Indian MORB, suggesting that it was ultimately derived from a source with a composition similar to the source of Indian MORB.

The primitive-mantle-normalized incompatible element patterns of the most enriched low-Ti glasses show enrichment in the moderately to highly incompatible elements, relative enrichments in Nb and Ta and relative depletions in K, Pb and Zr-Hf, general characteristics of HIMU-type lavas (Fig. 4). Below we will test the source and melt mixing models to see which can best explain the compositions of the low-Ti melts.

First, we test the source-mixing model (Fig. 4b) using the composition of primitive (MgO>10 wt%) HIMU-like lavas from the Rurutu Island, Austral archipelago, Central Pacific^34^, which have isotope compositions similar to the most LREE-enriched low-Ti rocks, to serve as the enriched end member. The model reproduces the general shape of the incompatible-element patterns and abundances of U, Nb, Ta and LREE in the primitive low-Ti Manihiki magmas well (Fig. 4b). Small discrepancies are however evident, primarily for fluid-mobile incompatible elements (Rb, Ba, U and Sr). A slightly better fit for the incompatible elements in the low-Ti Manihiki primary magmas can be achieved if the most enriched Macquarie Island ophiolite glasses^35^ (Supplementary Fig. 4a), which also have HIMU-like incompatible element compositions but distinct (Pacific-MORB-like) isotopic compositions, are assumed for the enriched end member. The addition of ∼0.06 to 1% of the Rurutu or Macquarie enriched end members to the UDM source can reproduce the full range of low-Ti Manihiki incompatible-element patterns. A similarly good fit to the Manihiki magmas can be obtained by modelling direct mixing of ultra-depleted melt from the UDM source and an enriched Rurutu or Macquarie melt (Supplementary Fig. 4b,c). The melt-mixing model, however, requires an order of magnitude more enriched melt (1–10%) to contribute to the Manihiki primary magmas than the source-mixing model. The melt-mixing model was therefore rejected as less probable due to the absence of correlations between major and trace elements in Manihiki glasses, expected from the relatively large mass fraction of the enriched melt required to fit the trace-element patterns. The origin of the magmas can thus be qualitatively attributed to melting ultra-depleted mantle metasomatized by the enriched melt component, present either cryptically or as a different lithology.

The possible isotopic composition of the enriched component was calculated by solving the general mixing equations for isotope ratios^30^ to fit the estimated mass fraction of the enriched component in the UDM obtained from trace element modelling and assuming Sr, Nd and Pb concentrations in the enriched component equal to the Rurutu melt used for the mixing model (see Fig. 4b). The estimated initial isotope composition of the enriched component is ^143^Nd/^144^Nd=0.51279±0.00001, ^206^Pb/^204^Pb=19.87±0.16, ^207^Pb/^204^Pb=15.66±0.03, ^208^Pb/^204^Pb=39.17±0.11, ^87^Sr/^86^Sr=0.70280±0.00007 (see Fig. 3, Supplementary Fig. 1, Supplementary Table 5). The end member ratios are very similar to those in the most-enriched low-Ti glass sample SO225DR12-3 with ^206^Pb/^204^Pb_(t)_=19.78, ^207^Pb/^204^Pb_(t)_=15.65, ^208^Pb/^204^Pb_(t)_=39.12, ^87^Sr/^86^Sr_(t)_=0.70282 and ^143^Nd/^144^Nd_(t)_=0.51279.

The enriched Manihiki end member has Sr-Nd-Pb isotopic ratios similar to lavas from sources with HIMU-type Ocean Island Basalt lavas from the Cook-Austral chain, such as those from Rurutu (young lavas) and Tubuai^34^,^36^ at 120 Myr ago (Fig. 3; Supplementary Fig. 1). Following the prevailing point of view, the HIMU-like enriched component may originate from recycled oceanic crust^37^.

On the basis of our new and previously published data, OJP and Manihiki Plateau, as well as possibly Hikurangi Plateau, were likely formed by melting of a compositionally heterogeneous plume source. The dominant plume component beneath the OJP and possibly the Hikurangi part of the original super-plateau (before breakup into individual plateau fragments) was the source of Kroenke/Kwaimbaita basement lavas^5^,^10^,^11^ (Fig. 5), which possibly represented a nearly primitive mantle source from the lower mantle^12^. The UDM component might also be present in the OJP source, but thus far has never been recognized in whole rock samples, but only as melt inclusions in olivine from one OJP Kroenke-type rock^16^. The dominant plume component beneath Manihiki appears to be the UDM metasomatized by HIMU-like melts. Together the UDM (mantle from which melt has been previously extracted) and enriched HIMU-like (recycled ocean crust) end members may represent relatively young, subducted Indian-MORB-like oceanic lithosphere, which stalled in the transition zone. Such oceanic lithosphere could have been entrained and recycled by the OJP/Manihiki/Hikurangi plume head as it upwelled from the lower mantle. Incorporation of the dense subducted ocean lithosphere could also have served to reduce the buoyancy of the upwelling plume head, such that it did not cause major uplift of the seafloor^38^. This could explain why the OJP, Manihiki and Hikurangi Plateaus remained largely submarine throughout their history on the seafloor. A piece of this entrained oceanic crust (eclogite) may still be located beneath OJP as proposed in a recent study^39^ to explain the fast shear wave velocity of 4.75 km s^−1^ observed beneath OJP. Regardless of the origin of the UDM and HIMU-like components, the composition of the mantle source below OJP (Kroenke/Kwaimbaita with minor UDM) and Manihiki (mainly UDM with some HIMU-like component) was largely different (Fig. 5). In conclusion, our results indicate that melting of incompatible-element ultra-depleted and subsequently re-enriched mantle is possible on a large-scale in intraplate settings during the formation of large igneous provinces, especially under young, thin oceanic lithosphere.

The presence of boninites in Archaean rock formations has been cited as evidence that subduction, and thus plate tectonics, initiated in the Archaean^40^. Recently, comparison of rock sequences including boninites from a modern arc system (Izu-Bonin-Mariana) and an Archaean greenstone belt in Canada suggested that subduction of oceanic crust has been operating since the Hadean^40^. High degrees of alteration, however, are common in Archaean rocks, affecting in particular major and mobile trace elements. The results of our study show that rocks with affinities to boninites in terms of major and trace elements (Fig. 2) can also originate in intraplate settings. Such melts may have been more abundant in earlier Earth history due to a hotter geotherm or enhanced plume-related magmatism. Thus, it is important to distinguish Archaean boninites that formed in subduction versus intraplate settings. The principal difference between intraplate boninite-like and most subduction-related boninites may be whether the Nb-Ta anomaly is positive or negative and if Nb/U normalized to N-MORB is greater or less than one. For example, in the Abitibi greenstone belt of Canada, some low-Ti rocks^41^ have trace element patterns very similar to those of the low-Ti Manihiki tholeiites (Supplementary Fig. 5). Thus, these Archaean sequences could possibly be plume-related unlike greenstones from the Nuvvuagittuq supracrustal belt in northern Quebec^42^ or Isua, Greenland^43^, which have arc-like boninite signatures with low Nb/La^40^,^42^. As a consequence, subduction-related Archaean boninites may not be as abundant as previously thought, placing some questions as to the exact initiation and on the extent of subduction, and thus of large-scale plate tectonics, in the early Earth. Future appraisal of Archaean mafic rocks may be able to exploit this distinction.

## Methods

### Sample material

**Figure 1:**
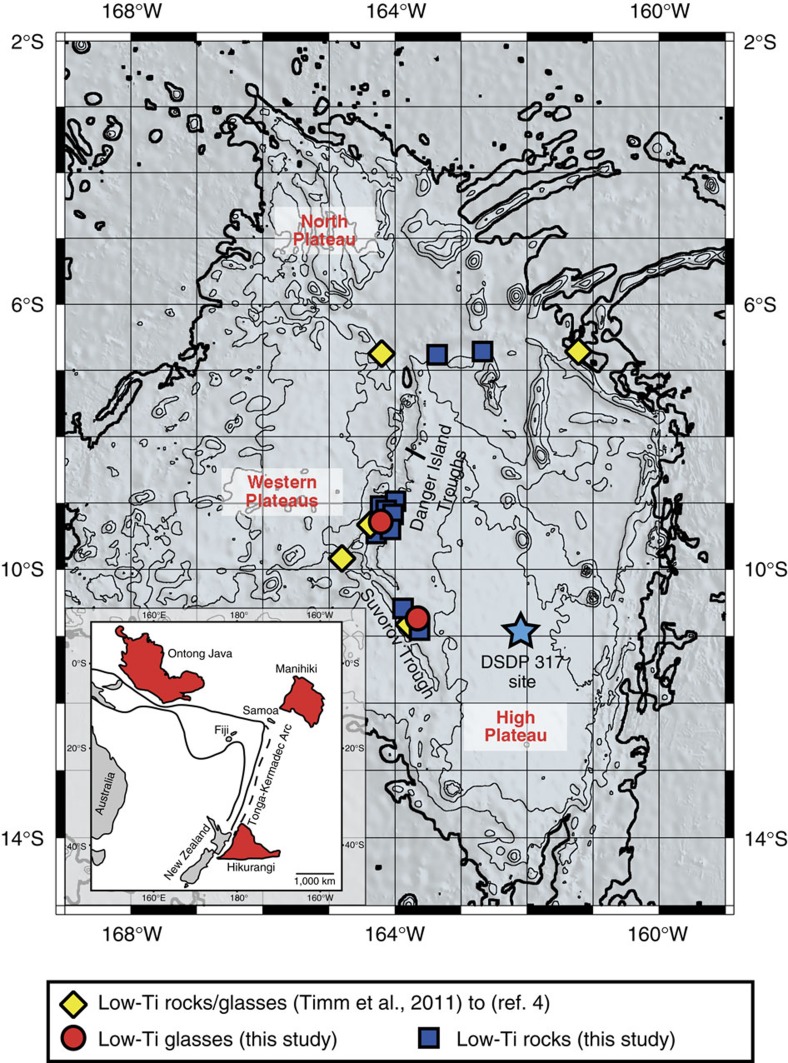
Bathymetric map of the Manihiki Plateau. Red circles show dredge sites of glass samples used for this study from the SO193 and SO225 expeditions. Yellow diamonds show additional dredge sites where low-Ti rock samples have been recovered and reported in ref. 4, and blue boxes show dredge sites where low-Ti rocks were recovered during the SO225 expedition, illustrating the large-scale distribution of low-Ti lavas at Manihiki. The contour lines denote depth intervals of 1,000 m with the thick black contour line showing a depth of 5,000 m below sea level. The map was created based on GEBCO bathymetry data^65^.

**Figure 2:**
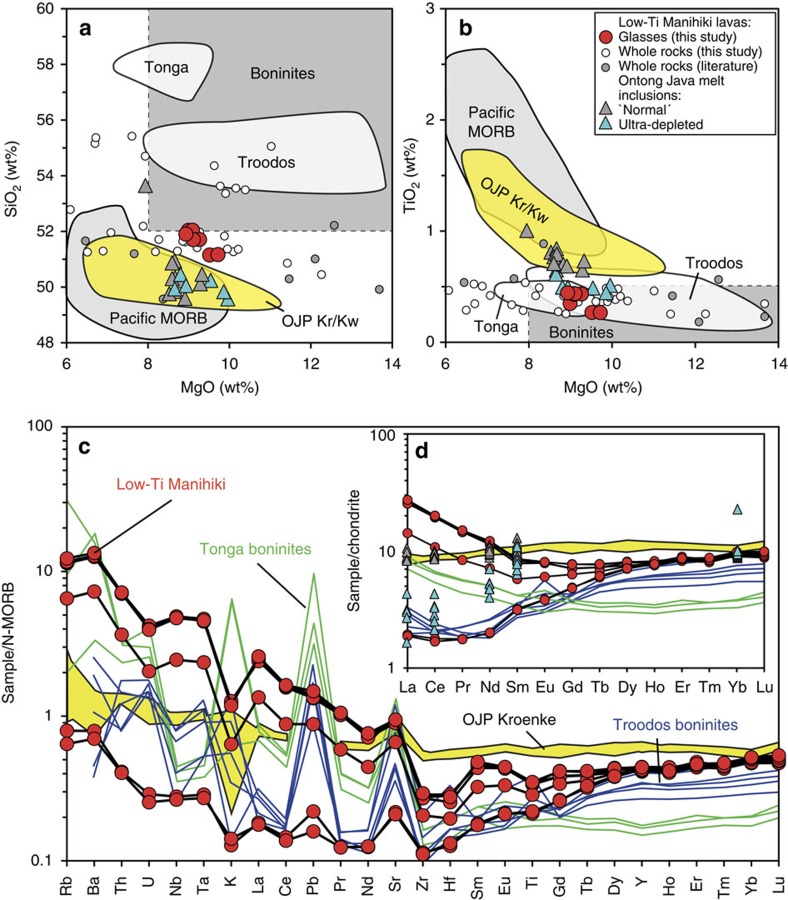
Selected major oxide and incompatible element compositions of low-Ti Manihiki rocks and glasses. (**a**) MgO versus SiO_2_ and (**b**) MgO versus TiO_2_ plots for low-Ti Manihiki glasses (red circles) and whole rock samples (white circles: this study, Supplementary Table 2; grey circles: data from refs 4, 13). All major element oxide data were normalized on a volatile-free basis to 100%. Compositional fields for Tonga and Troodos boninites^26^,^27^, Kroenke/Kwaimbaita OJP rocks and glasses^6^,^15^, melt inclusions from Ontong Java^16^ and Pacific MORB^66^ are shown for reference. Compositional range of boninites (>52 wt% SiO_2_, >8 wt% MgO and <0.5 wt% TiO_2_) is shown by grey field with dashed outer line^14^. (**c**) Incompatible element contents in low-Ti Manihiki glasses (black patterns), high-Ca boninites from Tonga (green patterns) and Troodos (blue patterns) normalized to N-MORB. (**d**) Rare earth elements in the same samples normalized to chondritic abundances. Most low-Ti glass samples from Manihiki have U-shaped patterns similar to boninites, reflecting in part re-enrichment of the most incompatible elements in a depleted source. Direct comparison of incompatible elements shows that boninite-like low-Ti Manihiki glasses do not show enrichment in fluid-mobile elements or negative Nb-Ta anomalies characteristic of boninites. Representative compositions for Troodos and Tonga boninites^67^,^68^ and OJP Kroenke-type rocks (OJP Kroenke; yellow field)^6^ are also shown. OJP inclusions^16^ in **d** have similar labelling as in **a**,**b**. N-MORB and chondrite compositions for normalization are from ref. 32.

**Figure 3:**
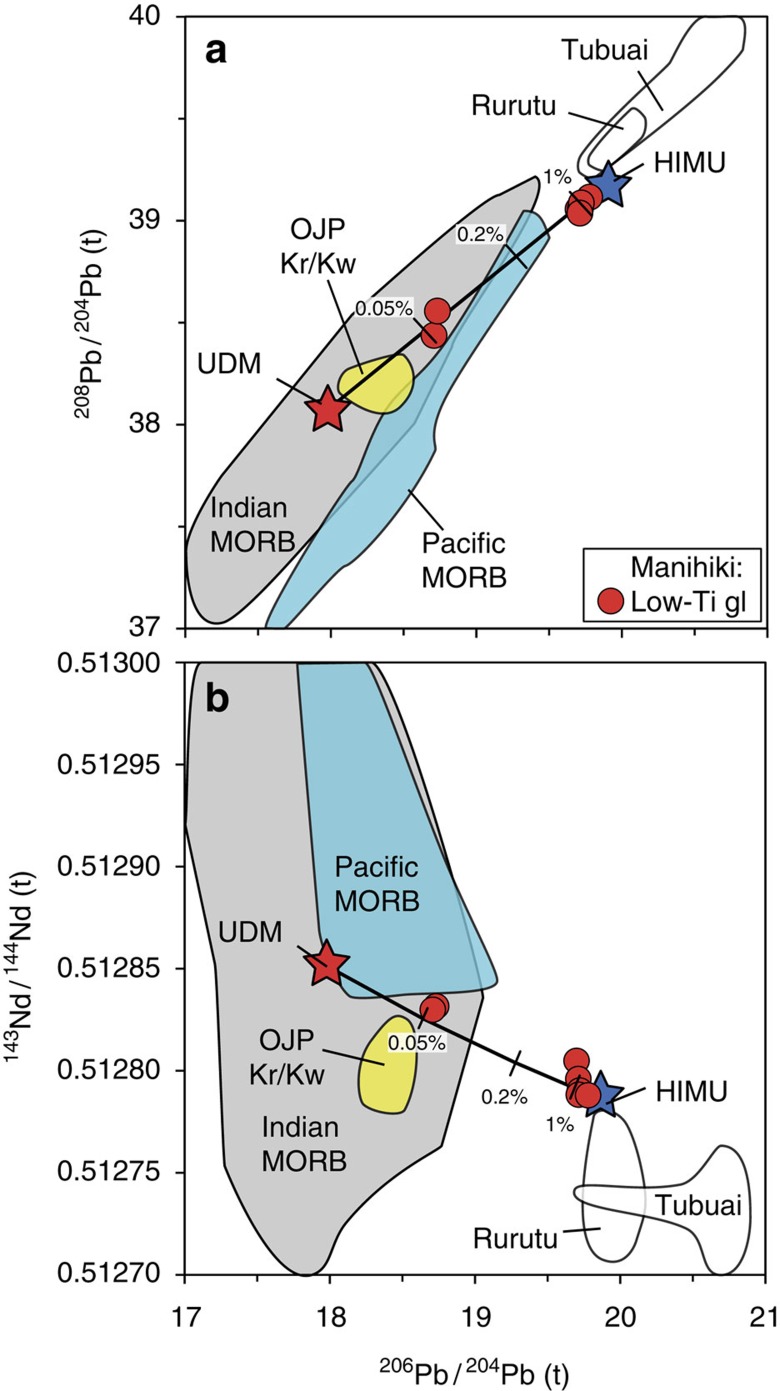
Radiogenic isotopic composition of low-Ti Manihiki rocks. Initial radiogenic isotope ratio plots (with correction for radiogenic ingrowth to 120 Myr ago) of (**a**) ^206^Pb/^204^Pb versus ^208^Pb/^204^Pb and (**b**) ^206^Pb/^204^Pb versus ^143^Nd/^144^Nd for low-Ti glasses. The stars represent the estimated UDM (red) and enriched (blue) end members for the Manihiki low-Ti tholeiites. The enriched melt end member for the Manihiki low-Ti tholeiites has characteristics similar to HIMU-like OIB lavas from Rurutu and Tubuai^69^ of the Cook-Austral Chain (see discussion, Supplementary Fig. 2 for calculation of the Manihiki-enriched end member). The black lines show mixing of UDM with variable amounts (indicated at tick marks in wt%) of the enriched OIB-like component represented by a mafic Rurutu lava sample from ref. 34. Note: ^143^Nd/^144^Nd ratio for intermediate sample SO225DR12-2 was not included for end member calculations (see ‘Sample material' in the ‘Methods' section). Isotope data for the Kroenke/Kwaimbaita field were taken from refs 10, 11. The MORB-field data^70^ were projected to 120 Myr using the following parent/daughter ratios from ref. 4: ^147^Sm/^144^Nd=0.25, ^87^Rb/^86^Sr=0.005, ^238^U/^204^Pb=10, ^235^U/^204^Pb=0.073, ^232^Th/^204^Pb=40. The Indian MORB^70^ field was projected to 120 Myr using calculated parent/daughter ratios based on the incompatible element composition of E-DMM from ref. 33. The fields for the Tubuai and young Rurutu lavas were also projected to 120 Myr ago and plotted based on compiled literature data and parent/daughter ratios from the calculated OIB source from ref. 36 and references therein.

**Figure 4:**
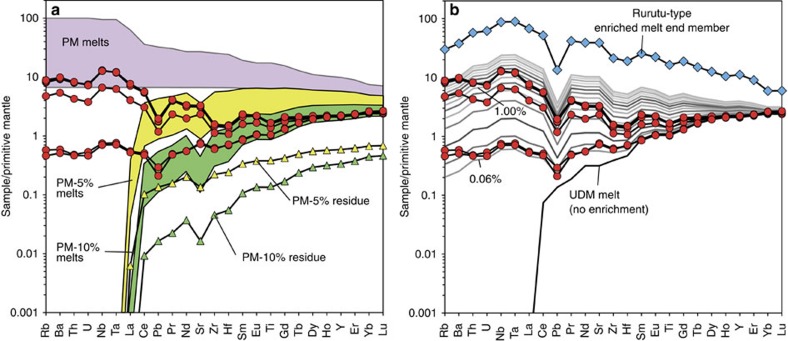
Two-stage mantle melting and source mixing to generate low-Ti primary Manihiki magmas. (**a**) Primitive-mantle-normalized multi-element plot of incompatible elements showing the compositions of primary low-Ti melts calculated from the compositions of Manihiki glasses (Supplementary Table 3; red circles); the ranges of melt compositions produced by pooled (aggregate) fractional melting from 1% to 15% of primitive mantle (PM) and of residual primitive mantle after 5% (PM-5%) and 10% (PM-10%) fractional melting; and residual mantle compositions after 5% and 10% previous melt extraction (yellow and green triangles, respectively). (**b**) The compositions of second stage melts produced by 8.7% pooled fractional melting of an UDM source. The UDM source was previously generated by 10.6% melt extraction from PM and subsequent enrichment/metasomatism with variable amounts (indicated in wt%) of melt corresponding in composition to the mafic Rurutu sample #RRT-B-30 from ref. 34. Details of the melt modelling are given in the discussion and input parameters in Supplementary Table 4.

**Figure 5:**
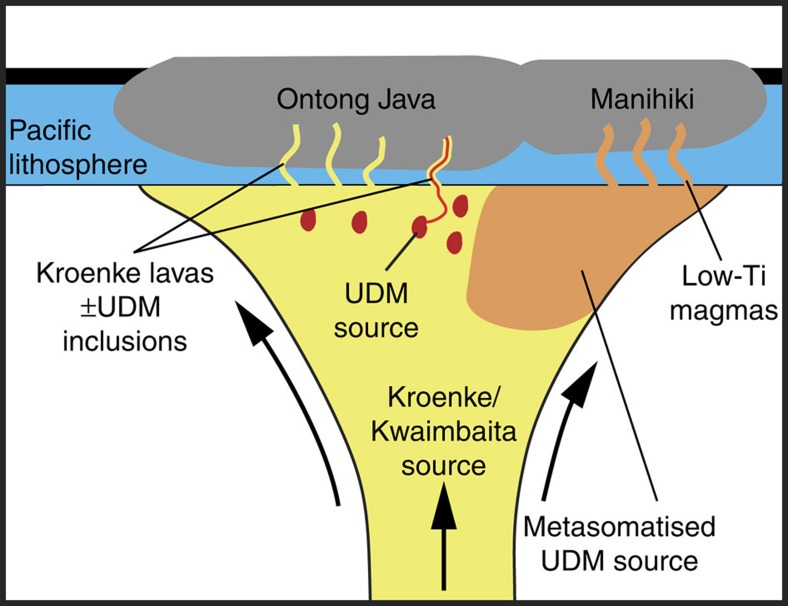
A schematic model for the formation of the Ontong Java and Manihiki Plateaus from a geochemically heterogeneous mantle-plume head. The presence of ultra-depleted melts at OJP and Manihiki (UDM) can be explained with a geochemically heterogeneous plume head containing different components beneath the two plateau segments. Beneath OJP, the dominant Kroenke/Kwaimbaita source component upwells and generates the dominant Kroenke/Kwaimbaita basement lavas with flat primitive-mantle-normalized incompatible element patterns. The presumed dominant source component beneath Manihiki is the UDM end member metasomatized by ≤1% HIMU-type melts. We propose that both components beneath Manihiki are derived through recycling of Indian-mantle-like oceanic lithosphere. Ultra-depleted melt inclusions in olivines in a Kroenke sample from OJP^16^ indicate that smaller proportions of a possibly common UDM source may also be present beneath the OJP.

Analysed samples were dredged during the R/V Sonne 225 expedition at different locations along the flanks of the Danger Island and Suvorov Troughs (Fig. 1). Detailed information about sampling locations and sample descriptions can be found in ref. 44. All the glass samples contain fresh and partly altered olivine crystals, up to 0.5 mm in size. One relatively fresh, vesicular, olivine-phyric basalt sample with fresh glass (sample SO225DR24-10) was recovered at the eastern flank of the Suvorov Trough, containing fresh olivine phenocrysts (0.25–1.00 mm size). Concentrations of mobile elements and oxides (e.g. Rb, Ba, U, Pb, K_2_O, P_2_O_5_) correlate with immobile elements such as Zr, Nb, La and Ce and thus do not appear to have been affected by seawater alteration. The glass samples from the SO193 expedition (ref. 4 and references therein) were re-analysed with electron micro-probe (EMP) and laser ablation inductively coupled plasma source mass spectrometry (LA-ICP-MS) using the same analysing conditions and standards for better data comparison. The sample SO225DR12-2 contained only small amounts of fresh glass material and thus it was not possible to replicate the radiogenic isotope composition. This sample shows slightly higher ^143^Nd/^144^Nd and ^87^Sr/^86^Sr ratios compared with the more-enriched glass sample compositions, plotting slightly off the estimated correlation line and therefore data for this sample have not been used to estimate the end member compositions.

Whole-rock samples from the SO225 and SO193 cruise are fine-grained and show different degrees of seawater alteration from fresh to strongly altered samples (loss of ignition (LOI): 1–13 wt%, see Supplementary Table 2).

### Electron microprobe analysis

Major elements, S and Cl in Manihiki glasses were analysed with a JEOL JXA 8200 wavelength dispersive microprobe at GEOMAR (Kiel, Germany) using a defocused to 5 μm electron beam, 15 kV acceleration voltage, 6 nA beam current and other conditions as in ref. 45. All elements except Cl, S and F were calibrated using basaltic reference glasses USNM 111240/52 VG-2 and USNM 113498/1 VG-A99 (ref. 46). S and Cl were calibrated using scapolite USNM R6600-1 (ref. 46) and F on rhyolite glass KN-18 (ref. 47). The data accuracy was monitored using basaltic glasses USNM 111240/52 VG-2 and USNM 113498/1 VG-A99 (Supplementary Table 6a) and rhyolite glass USNM 72854 VG568 (ref. 46). The data reported in Supplementary Table 1 represent average compositions obtained from 20 to 25 analyses performed on 10–12 glass shards for every sample. All glasses were homogeneous within the precision of analyses estimated from replicate measurements of reference glasses.

### X-ray fluorescence

Whole-rock samples (powders) were analysed for major elements by X-ray fluorescence spectrometry (XRF) on fused pellets using a Magix Pro PW 2540 XRF at the Institute of Mineralogy and Petrography at the University of Hamburg, Germany. International reference samples JGB-1, JB-3, JB-2, JA-3 and JG-3 provided by the Geological Survey of Japan (https://gbank.gsj.jp/geostandards/gsj1mainj.html)^48^ were measured along with the samples (Supplementary Table 6b).

### Laser ablation inductively coupled plasma mass spectrometry

Trace element concentrations were determined by LA-ICP-MS using an Agilent 7500s quadrupole instrument coupled with a GeoLas Pro 193 nm excimer laser system at the Institute of Geosciences (IfG), Kiel University. Sample mounts were mounted in a two-volume ablation cell^49^, and the ablated sample aerosol was transported with helium as the carrier gas and mixed with argon prior to introduction into the spectrometer. Glass shards were analysed with 60–80 μm crater size, 10 J cm^−2^ laser energy and 10 Hz laser frequency. The total analysis time for each single analysis was set to 80 s (20 s background and 60 s counting time during laser ablation). The measured intensities were converted to concentrations in the Glitter software by using ^43^Ca as internal standard and Ca concentrations from EMPA. The SRM NIST612 standard^50^ was used for initial calibration. Matrix correction was applied by using data for KL2-G reference glass^51^. Reference glasses GOR128-G and BCR-2G were analysed as unknown, and the data are listed in Supplementary Table 6c. Consistency between EMPA and LA-ICP-MS data was checked using Ti contents, which agreed within 10% rel. between the two techniques. Analytical precision for multiple analyses was typically better than 3% rel. for most elements. Further details of the setup and calibration strategy can be found in ref. 52. The LA-ICP-MS data in Supplementary Table 1 are average trace element concentrations obtained from three to five spots for every sample. In addition, whole-rock powder of the sample SO225DR24-10 was analysed for trace elements using an Agilent 7500cs ICP-MS instrument following acid digestion as described in ref. 53. The trace element data generated by laser ablation ICP-MS on glasses and solution ICP-MS on the bulk sample show excellent agreement on p.p.m. to sub-p.p.m. concentrations, except for elements easily affected in bulk rocks by seawater alteration (for example, Cs, Sb, Rb, U). Elements concentrated in olivine and chromium spinel (Ni, Cr) have systematically higher concentrations in the whole-rock solution ICP-MS data.

### Secondary ion mass spectrometry

Concentrations of H_2_O were determined using the CAMECA IMS 1280 HR2 instrument at CRPG (Nancy, France), following the approach taken by ref. 54. The samples (previously analysed for major element contents by EMPA) were carefully re-polished to remove any residual carbon from the carbon-coating process (finishing with the 0.25 μm grain size Al_2_O_3_ suspension), ultrasonically cleaned, removed from the epoxy mounts and then remounted by pressing them into indium metal. The samples were sputtered with a 10 kV ^133^Cs^+^ primary beam with a beam current of 0.8–1.5 nA focused to 5–10 μm spots and rastered to 20 × 20 μm. A normal-incidence electron flood gun was used to compensate for sample charge. A field aperture of ∼1,000 μm was used to eliminate secondary ion signal that may come from spot margins due to residual carbon surviving the cleaning process. A mass resolving power (MRP) of ∼5,000, enough to resolve ^17^O from ^16^OH and ^29^SiH from ^30^Si peaks, was applied. The ^12^C^−^ (6 s), ^16^OH^−^ (4), ^27^Al^−^ (2 s) and ^30^Si^−^ (2 s) ions were counted after 300 s pre-sputtering during 12 cycles. To establish the calibration curves, reference glasses (experimental glasses M9, M15 and M47 from the Institut für Mineralogie, Leibniz Universität Hannover^55^, as well as natural standard reference glasses ETNA II-6, ETNA II-7 and ALV 981 R23 (ref. 22 and references therein)) with CO_2_=0−365 p.p.m., H_2_O=0.0−1.5 wt.% and SiO_2_=48−50 wt.% were analysed. Olivine was also analysed together with the glasses to account for H_2_O and CO_2_ background.

### Thermal ionization mass spectrometry

Sr-Nd-Pb isotope analyses were carried out at GEOMAR Helmholtz Centre for Ocean Research, Kiel, Germany by thermal ionization mass spectrometry (TIMS) following the methods outlined in ref. 56. In brief, before sample dissolution in hot HF-HNO_3_, approximately 100–250 mg hand-picked glass (125–250 μm) and whole rock (250–500 μm) chips were leached in warm 2 N HCl at 70 °C for 1 h and subsequently triple-rinsed in 18 MΩ water to minimize the effects of alteration and sample handling. Ion chromatography followed established standard procedures^57^,^58^. Pb isotope analyses were performed on a Finnigan MAT 262 RPQ^2+^ using the Pb double-spike (DS) technique of ref. 59. DS-corrected NBS981 values obtained over the course of the study are ^206^Pb/^204^Pb=16.9416±0.0027, ^207^Pb/^204^Pb=15.4990±0.0027, ^208^Pb/^204^Pb=36.7247±0.0069, ^207^Pb/^206^Pb=0.914849±0.000043 and ^208^Pb/^206^Pb=2.16772±0.00013 (*n*=84; 2σ external reproducibility) and they compare well with published double- and triple-spike data^60^,^61^,^62^,^63^,^64^. Nd and Sr isotope ratios were measured on a Thermo Fisher TRITON TIMS. Within-run mass bias correction used ^146^Nd/^144^Nd=0.7219 and ^86^Sr/^88^Sr=0.1194. NBS987 and La Jolla reference material was measured five to six times each turret to obtain a normalization value relative to our preferred values of ^87^Sr/^86^Sr=0.710250 and ^143^Nd/^144^Nd=0.511850, respectively. The normalization value was then applied to the sample data of each turret. Over the course of the study, the long-term machine drift compensated external 2σ uncertainties (2SD) are ±0.000011 (*n*=25) for NBS987 and ±0.000007 (*n*=33) for La Jolla. Within-run 2 s.e. errors are equal or smaller than 2 s.d. of the reference materials. Total chemistry blanks were 10 to 30 pg for Pb and below 150 pg for Sr and Nd and thus negligible. Replicate analyses by means of a second sample digest were carried out for SO225 ROV-3-6 whole-rock chips. While Sr and Nd isotope ratios are reproduced within 2 s.d. of the standards, only ^207^Pb/^204^Pb of the Pb isotope ratios matches this criteria. The limited reproducibility of Pb isotope ratios involving ^206^Pb and ^208^Pb is ascribed to the combined effects of (i) seawater interaction during low temperature alteration that can lead to a heterogeneous enrichment of uranium causing variable degrees of ^206^Pb ingrowth over time and (ii) Pb removal at high temperatures leading to variable U/Pb and Th/Pb and thus ^206^Pb and ^208^Pb ingrowth. The slight variations caused by alteration, however, do not affect the overall scientific interpretations and conclusions derived from the Pb isotope data.

### Data availability

The authors declare that all data supporting the findings of this study are available within the paper and its Supplementary Information files.

## Additional information

**How to cite this article:** Golowin, R. *et al*. Boninite-like intraplate magmas from Manihiki Plateau require ultra-depleted and enriched source components. *Nat. Commun.* **8,** 14322 doi: 10.1038/ncomms14322 (2017).

**Publisher's note:** Springer Nature remains neutral with regard to jurisdictional claims in published maps and institutional affiliations.

## Supplementary Material

Supplementary Information Supplementary Figures, Supplementary Tables and Supplementary References

Supplementary Data 1 Supplementary Tables in Excel format
