# Supplementary material for: Boninite-like intraplate magmas from Manihiki Plateau require ultra-depleted and enriched source components
Source: Nat Commun. 2017 Feb 9;8:14322. doi: 10.1038/ncomms14322 (PMC5309764; doi:10.1038/ncomms14322)
Supplement: Supplementary Information — Supplementary Figures, Supplementary Tables and Supplementary References [file ncomms14322-s1.pdf]

SUPPLEMENTARY INFORMATION

Figures:

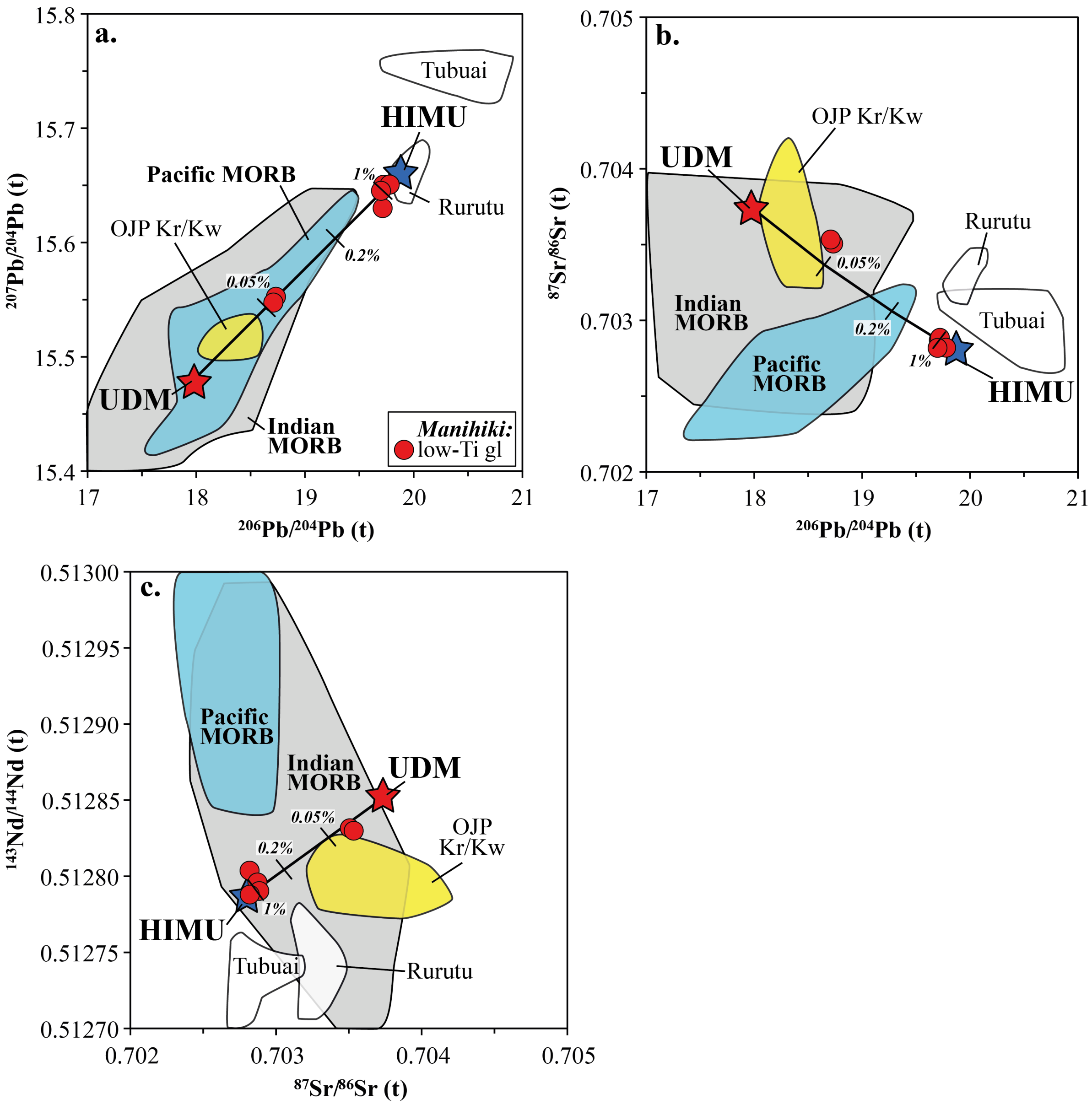

Supplementary Fig. 1. Radiogenic isotopic composition of low-Ti Manihiki rocks. Initial radiogenic isotope ratio plots (with correction for radiogenic ingrowth to 120 Ma) of (**a**) $^{206}Pb/^{204}Pb$ vs. $^{207}Pb/^{204}Pb$, (**b**) $^{206}Pb/^{204}Pb$ vs. $^{87}Sr/^{86}Sr$ and (**c**) $^{87}Sr/^{86}Sr$ vs. $^{143}Nd/^{144}Nd$ for low-Ti glasses. The stars represent the estimated Manihiki UDM (red) and enriched (blue) end members for the low-Ti tholeiites. The enriched melt end member for the Manihiki low-Ti tholeiites has characteristics similar to HIMU-like OIB lavas from Rurutu and Tubuai[1] of the Cook-Austral Chain (see discussion text and Supplementary Fig. 2 for calculation of the

Manihiki enriched end member). The black lines show mixing of UDM composition with the estimated enriched melt end member with different mixing proportions using Pb, Sr and Nd concentrations of the mafic Rurutu sample (#RRT-B-30) from ref. [2] used as metasomatising melt. Note: the Sr and Nd isotope ratios of intermediate sample SO225DR12-2 were not used for calculation of end members (see sample material in methods). Isotope data for the Kroenke/Kwaimbaita fields were taken from refs. [3,4]. The MORB-field data[5] were projected to 120 Ma using the following parent/daughter ratios from ref. [6]: $^{147}Sm/^{144}Nd=0.25$, $^{87}Rb/^{86}Sr=0.005$, $^{238}U/^{204}Pb=10$, $^{235}U/^{204}Pb=0.073$, $^{232}Th/^{204}Pb=40$. The Indian MORB[5] fields were projected to 120 Ma using calculated parent/daughter ratios based on the incompatible element composition of E-DMM from ref. [7]. The fields for the Tubuai and young Rurutu lavas were also projected to 120 Ma and plotted based on compiled literature data and parent/daughter ratios from the calculated OIB source from ref. [8] and references therein.

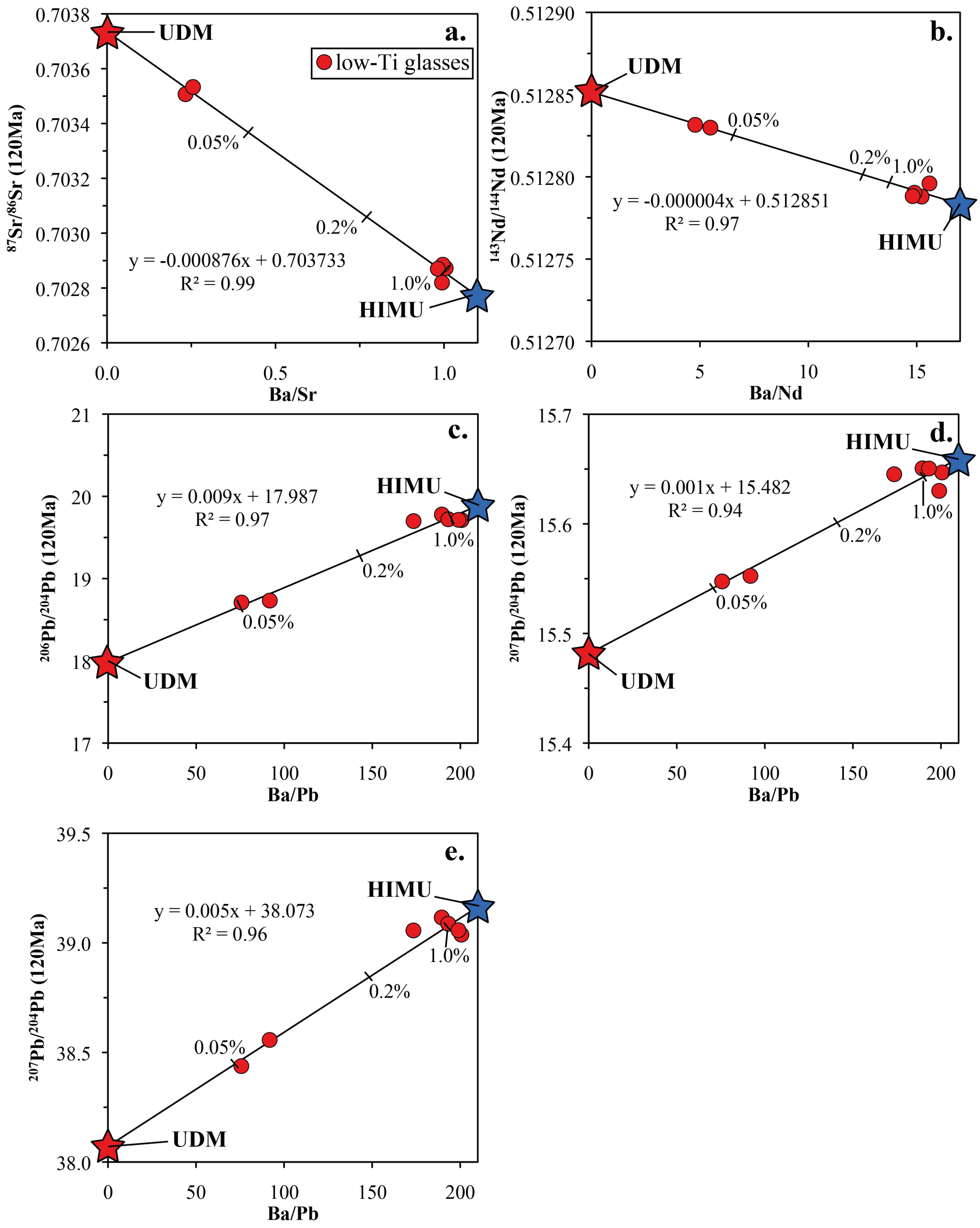

Supplementary Fig. 2. Incompatible element and radiogenic isotope ratio systematics of low-Ti Manihiki glasses.

The low-Ti Manihiki glass samples are shown as red circles on panels **a-e** and estimated UDM and enriched HIMU-like components as red and blue stars, respectively. Assuming linear correlations, which imply two-component mixing relationships within the low-Ti glasses allows estimation of the compositions of the ultra-depleted (red star) and enriched (blue star) end members. The composition of sample SO225DR12-2 was not used for the estimation of $^{87}Sr/^{86}Sr$ and $^{143}Nd/^{144}Nd$ ratios in the ultra-depleted and enriched end members.

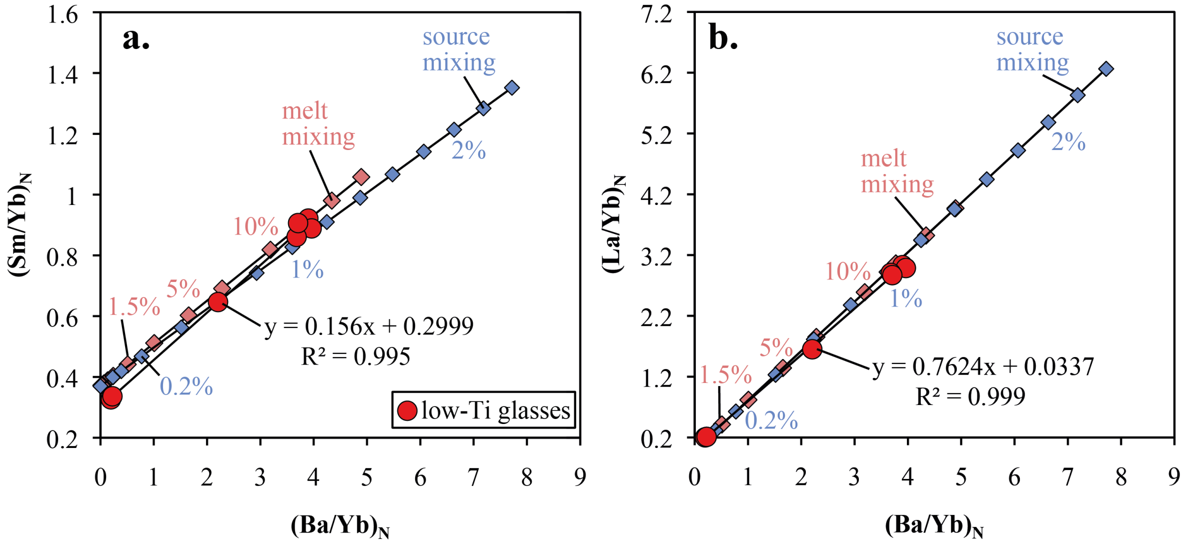

Supplementary Fig. 3. Correlation of incompatible element ratios in low-Ti glasses. The highly statistically significant linear correlations of low-Ti glasses (red circles on panels **a-b**) imply either melt mixing or melting of heterogeneously metasomatised source[9] (see discussion in text).

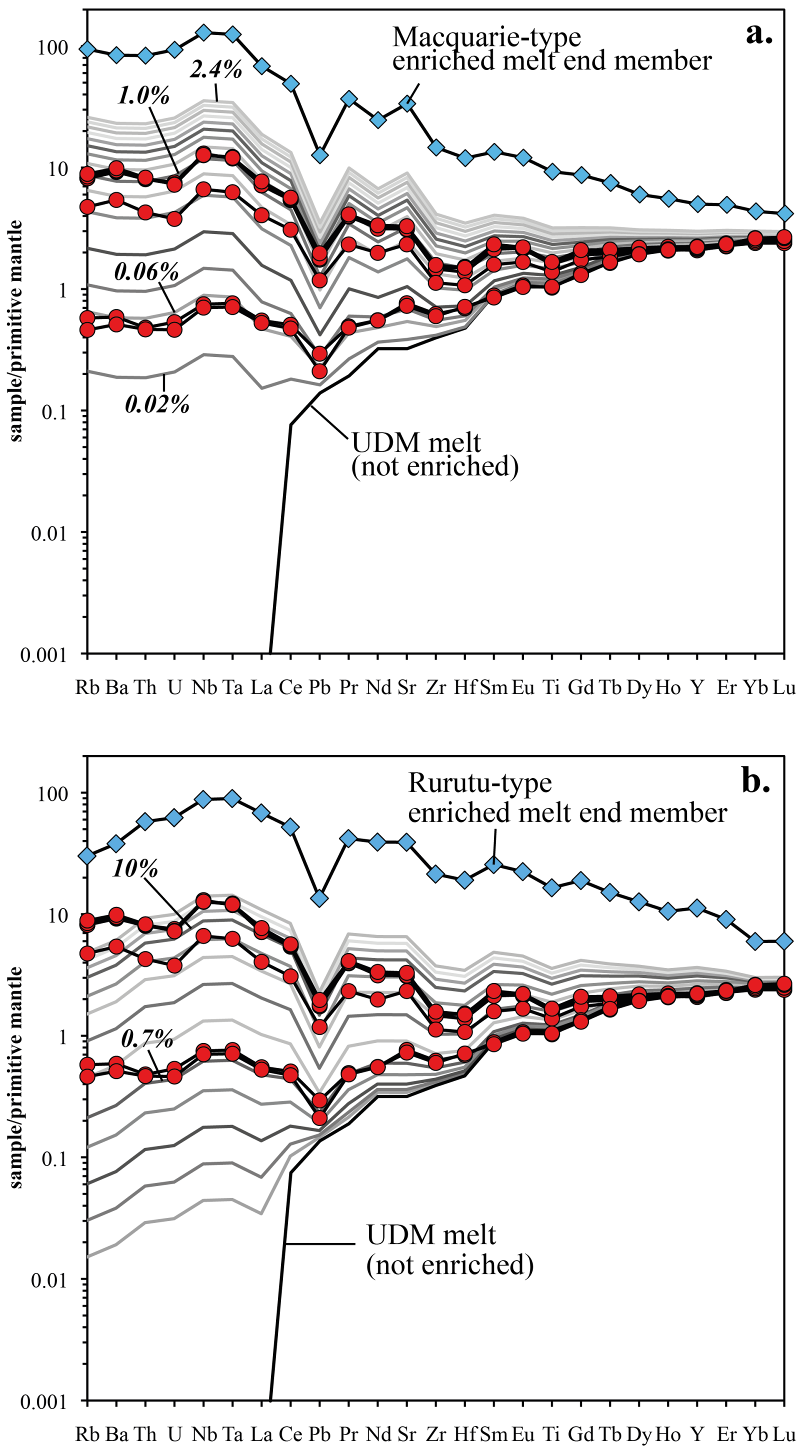

a.
Macquarie-type
enriched melt end member
2.4%
1.0%
0.06%
0.02%
UDM melt
(not enriched)
sample/primitive mantle
100
10
1
0.1
0.01
0.001
Rb Ba Th U Nb Ta La Ce Pb Pr Nd Sr Zr Hf Sm Eu Ti Gd Tb Dy Ho Y Er Yb Lu
b.
Rurutu-type
enriched melt end member
10%
0.7%
UDM melt
(not enriched)
sample/primitive mantle
100
10
1
0.1
0.01
0.001
Rb Ba Th U Nb Ta La Ce Pb Pr Nd Sr Zr Hf Sm Eu Ti Gd Tb Dy Ho Y Er Yb Lu

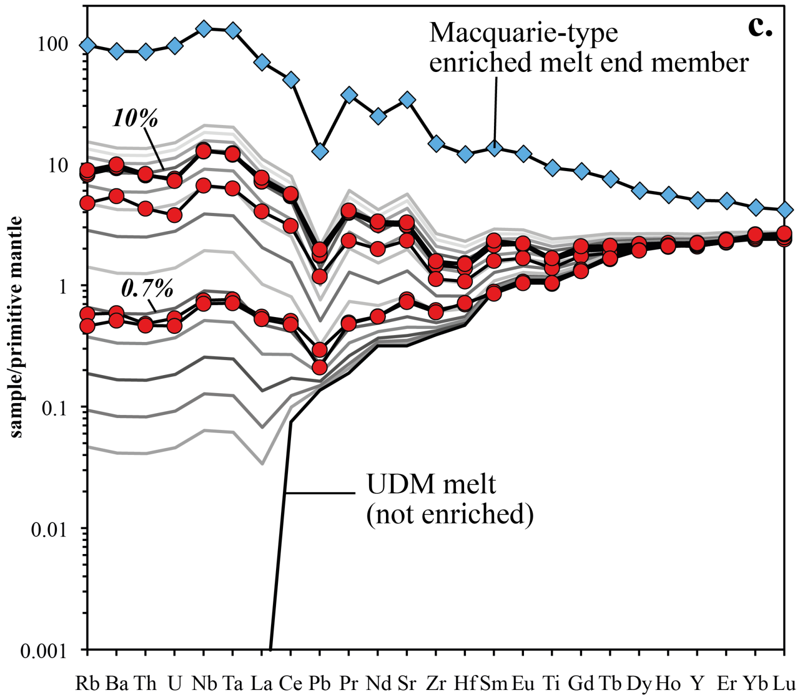

Supplementary Fig. 4. Mixing model for the generation of low-Ti magmas at Manihiki. (**a**) The compositions of melts produced by 8.7% of pooled fractional second stage melting of UDM source enriched by variable amounts (indicated in wt%) of Macquarie melt (using composition of glass sample 47963[10]). **(b-c)** The compositions of melts produced by mixing of ultra-depleted melt derived through 8.7% melting of residue after 10.6 % fractional melting of primitive mantle and melt removal with (**b**) enriched Rurutu-type melt (sample #RRT-B-30[2]) and (**c**) enriched Macquarie melt (glass sample #47963[10]). Details for the modelling are given in the discussion and input parameters in Supplementary Table 4.

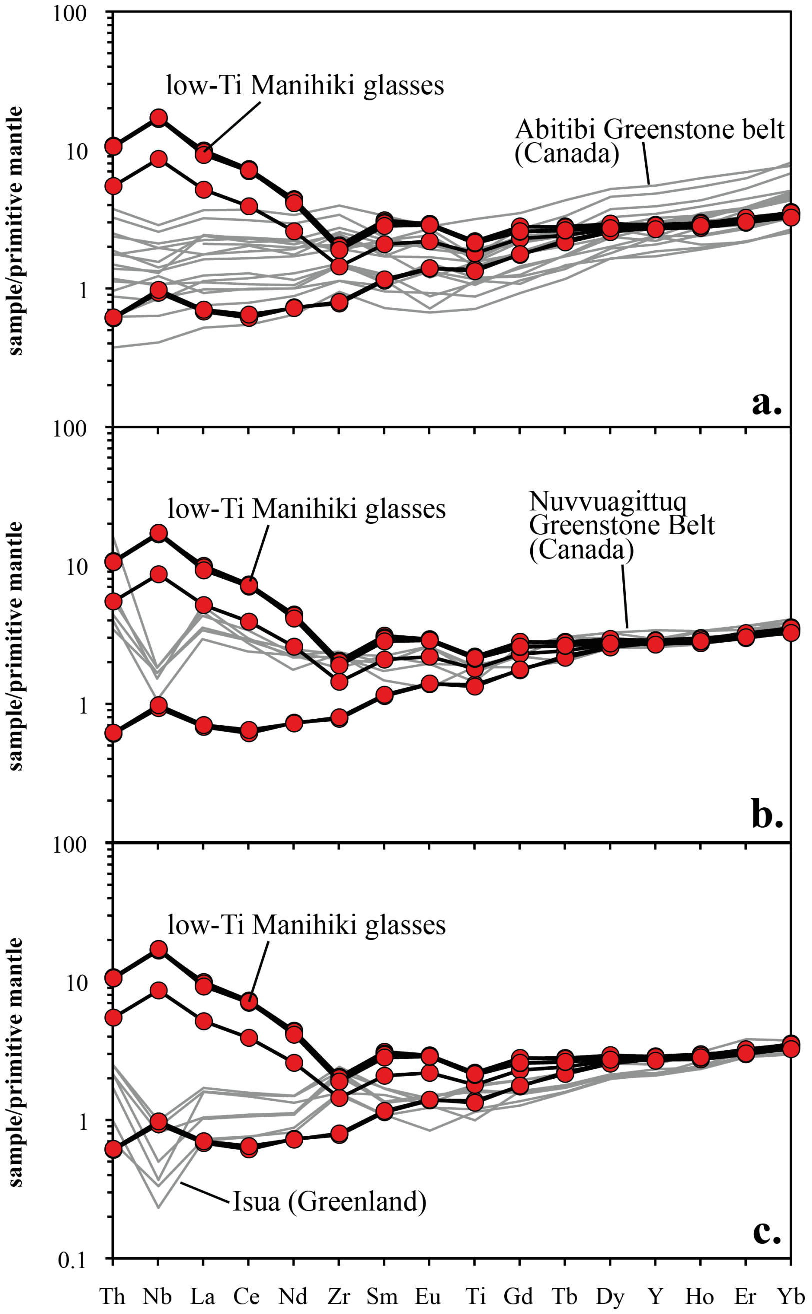

Supplementary Fig. 5. Comparison of low-Ti Manihiki glasses with Archean boninites. Primitive-mantle[11] normalized multi-element plots for low-Ti glasses from Manihiki with (**a**) Archean low-Ti, boninitic rocks from the Abitibi Greenstone Belt, Canada[12], (**b**) the Nuvvuagittuq supracrustal belt in northern Quebec[13] and (**c**) the Isua Greenstone Belt, Greenland[14]. Low-Ti rocks from Abitibi have similar patterns to the most depleted low-Ti Manihiki tholeiites, whereas boninitic rocks from Isua and Nuvvuagittuq exhibit low Nb/La ratios, most likely indicating a subduction-related origin.

**$^{206}Pb/^{204}Pb(t)$**[6]	19.714	19.721	19.711	19.699	19.779	18.709	18.732
**$^{207}Pb/^{204}Pb(t)$**[6]	15.630	15.650	15.647	15.645	15.651	15.547	15.553
**$^{208}Pb/^{204}Pb(t)$**[6]	39.058	39.087	39.037	39.056	39.116	38.438	38.557
**$^{87}Sr/^{86}Sr(t)$**[6]	0.702870	0.702885	0.702872	0.702818	0.702820	0.703533	0.703507
**$^{143}Nd/^{144}Nd(t)$**[6]	0.512788	0.512790	0.512796	0.512804	0.512788	0.512830	0.512832
**εNd(t)**[6]	5.94	5.98	6.09	6.26	5.94	6.76	6.79
**εNd**	5.05	5.01	5.13	5.66	5.03	8.57	8.74

Notes:
[1] analysed with SIMS
[2] molar Mg/Mg+Fe ratio
[3] FTIR data from Timm et al., 2011
[4] Analysed with XRF for major elements and solution ICP-MS for trace elements
[5] Double spike Pb data
[6] t=120Ma
[7] Isotopes analysed on whole rock chips
[8] isotope data from Timm et al., 2011 except for Sr isotope ratio in sample SO193DR26-1
N/A - not analysed
note: isotope data for sample SO225DR24-10 is from whole rock analyses

Supplementary Table 2: Major element composition of dredged low-Ti Manihiki rocks

Sample #	SO193DR18-3	SO225DR5-1	SO225DR7-1	SO225DR12-1	SO225DR12-4	SO225DR12-5	SO225DR12-7	SO225DR12-8	SO225DR12-12	SO225DR13-1
SiO_2	49.39	49.36	50.63	50.20	48.61	41.17	48.95	50.17	49.26	49.01
TiO_2	0.25	0.44	0.36	0.48	0.46	0.31	0.42	0.41	0.40	0.39
Al_2O_3	13.95	16.41	14.63	15.15	14.89	12.87	16.48	16.15	15.35	14.18
Fe_2O_3	9.48	10.41	9.80	9.54	7.60	11.63	6.10	8.45	8.35	9.43
MnO	0.16	0.08	0.16	0.13	0.09	0.16	0.08	0.10	0.12	0.15
MgO	11.08	5.68	9.05	8.01	9.25	12.09	8.72	6.83	8.43	9.48
CaO	12.23	8.37	12.75	12.33	11.51	8.47	11.60	12.70	12.17	11.68
Na_2O	1.36	2.70	1.93	1.73	1.83	1.69	1.97	2.04	1.73	1.55
K_2O	0.13	1.06	0.11	0.40	0.22	1.04	0.40	0.47	0.34	0.41
P_2O_5	0.03	0.04	0.03	0.06	0.08	0.18	0.07	0.08	0.06	0.07
SO_3	0.00	0.00	0.02	0.00	0.00	0.01	0.00	0.00	0.00	0.00
L.O.I	1.27	5.10	0.96	1.49	4.22	8.64	4.40	2.10	2.78	3.03
SUM	99.33	99.65	100.43	99.52	98.76	98.26	99.19	99.50	98.99	99.38

Sample #	SO225DR13-2	SO225DR13-3	SO225DR13-4	SO225DR16-1	SO225DR16-5	SO225DR16-10	SO225DR17-2	SO225DR17-3	SO225DR18-1	SO225DR18-2
SiO_2	49.23	48.91	48.59	50.56	47.41	47.30	47.06	46.58	51.52	50.66
TiO_2	0.40	0.37	0.40	0.32	0.47	0.40	0.45	0.46	0.35	0.35
Al_2O_3	14.11	13.86	15.07	13.86	16.85	12.59	15.84	15.32	14.55	14.55
Fe_2O_3	9.34	9.92	10.37	10.92	10.54	10.96	11.62	11.44	8.58	8.90
MnO	0.16	0.15	0.14	0.07	0.06	0.03	0.02	0.02	0.10	0.11
MgO	9.78	9.62	7.74	6.12	6.36	9.46	5.70	6.74	9.11	9.39
CaO	11.86	11.72	11.09	5.27	8.68	1.13	1.15	1.25	9.24	9.79
Na_2O	1.54	1.44	1.59	3.11	2.32	2.60	1.87	2.10	1.86	1.75
K_2O	0.31	0.34	0.74	1.88	0.68	2.44	2.64	2.26	0.28	0.29
P_2O_5	0.06	0.06	0.08	0.29	0.11	0.09	0.12	0.12	0.03	0.03
SO_3	0.00	0.00	0.00	0.02	0.00	0.00	0.00	0.00	0.00	0.00
L.O.I	2.75	2.80	3.59	6.86	6.00	11.76	12.76	12.71	3.89	3.77
SUM	99.54	99.19	99.40	99.28	99.48	98.76	99.23	99.00	99.51	99.59

Sample #	SO225DR18-4	SO225DR18-12	SO225DR19-1	SO225DR24-2	SO225DR24-3	SO225DR24-10	SO225DR27-2	SO225DR27-6	SO225DR27-7
SiO_2	50.83	49.86	47.57	49.71	49.63	48.72	50.43	49.00	52.00
TiO_2	0.35	0.35	0.43	0.27	0.25	0.25	0.28	0.28	0.32
Al_2O_3	14.48	14.53	15.18	14.97	14.37	13.81	16.09	16.30	14.10
Fe_2O_3	8.77	8.62	11.00	9.97	9.93	9.76	8.23	10.35	9.14
MnO	0.11	0.23	0.09	0.14	0.15	0.15	0.12	0.11	0.11
MgO	9.25	9.67	9.01	8.44	8.55	11.83	7.60	6.21	7.12
CaO	9.62	6.89	1.24	12.49	12.56	11.69	12.52	11.87	8.63
Na_2O	1.93	2.79	2.24	1.48	1.42	1.16	1.64	1.72	1.81
K_2O	0.28	1.09	3.04	0.41	0.23	0.16	0.47	0.72	1.44
P_2O_5	0.03	0.03	0.12	0.03	0.04	0.02	0.07	0.07	0.06
SO_3	0.00	0.00	0.00	0.00	0.00	0.04	0.00	0.00	0.00
L.O.I	3.77	5.10	8.94	1.83	2.28	1.21	2.54	3.13	4.98
SUM	99.42	99.16	98.86	99.74	99.41	98.80	99.99	99.76	99.71

Supplementary Table 3: Composition of calculated primary low-Ti magmas

major elements (in wt%)[1]	SO193DR26-1	SO193DR26-3	SO193DR26-10	SO225DR12-2	SO225DR12-3	SO225DR24-1	SO225DR24-10
**SiO_2**	49.56	49.47	49.84	50.14	50.01	49.43	49.16
**TiO_2**	0.35	0.35	0.36	0.29	0.36	0.22	0.22
**Al_2O_3**	11.91	11.92	12.20	12.72	12.29	12.94	12.54
**Fe_2O_3**	0.17	0.18	0.18	0.14	0.18	0.11	0.11
**FeO**	9.04	9.10	8.81	8.60	8.81	8.85	9.08
**FeO_t**	9.21	9.28	8.99	8.74	8.98	8.96	9.19
**MnO**	0.15	0.17	0.17	0.15	0.14	0.14	0.16
**MgO**	16.79	16.96	16.22	15.67	16.22	16.25	16.82
**CaO**	10.49	10.35	10.68	10.95	10.56	10.80	10.70
**Na_2O**	1.33	1.31	1.34	1.23	1.25	1.22	1.17
**K_2O**	0.15	0.14	0.15	0.07	0.14	0.02	0.02
**P_2O_5**	0.06	0.05	0.06	0.03	0.05	0.03	0.02
**T_{liq}**	1385	1388	1373	1361	1373	1373	1385
**T_p**	1503	1507	1489	1476	1489	1490	1504
trace elements (in ppm)[2]							
**Rb**	5.18	5.18	5.37	3.02	5.63	0.37	0.29
**Ba**	64.3	65.0	66.3	37.9	69.1	4.11	3.58
**Th**	0.68	0.69	0.69	0.36	0.70	0.04	0.04
**U**	0.15	0.16	0.15	0.08	0.15	0.01	0.01
**Nb**	9.01	9.10	9.30	4.72	9.06	0.53	0.50
**Ta**	0.50	0.50	0.49	0.26	0.49	0.03	0.03
**La**	4.90	5.05	4.91	2.79	5.28	0.38	0.36
**Ce**	9.60	9.68	9.86	5.47	10.0	0.90	0.84
**Pb**	0.32	0.34	0.33	0.22	0.36	0.05	0.04
**Pr**	1.09	1.10	1.10	0.64	1.14	0.14	0.13
**Nd**	4.34	4.36	4.25	2.69	4.54	0.75	0.75
**Sr**	65.5	65.2	65.9	49.2	69.5	16.1	15.4
**Zr**	16.5	16.9	16.4	12.6	17.7	6.98	6.70
**Hf**	0.47	0.47	0.43	0.33	0.46	0.21	0.22
**Sm**	1.00	0.97	0.94	0.71	1.04	0.39	0.38
**Eu**	0.36	0.36	0.36	0.28	0.37	0.18	0.17
**Ti**	2076	2085	2127	1800	2174	1336	1357
**Gd**	1.14	1.23	1.16	1.04	1.25	0.80	0.78
**Tb**	0.21	0.22	0.21	0.20	0.23	0.18	0.18
**Dy**	1.58	1.60	1.51	1.51	1.61	1.43	1.42
**Ho**	0.35	0.35	0.35	0.34	0.37	0.35	0.34
**Y**	9.57	9.85	9.48	9.72	10.2	9.89	10.1
**Er**	1.08	1.14	1.09	1.09	1.13	1.12	1.13
**Yb**	1.22	1.25	1.18	1.21	1.25	1.30	1.29
**Lu**	0.19	0.18	0.18	0.18	0.18	0.19	0.20

Notes:

[1] calculated with PRIMELT2 model from Herzberg & Asimow (2008)

[2] calculated based on the relative difference of the Al_2O_3 content between the measured glass composition and the calculated primary melt

Supplementary Table 4: Input parameters for trace element mantle melting and mixing modelling

Elements ppm	Inital mantle source PM[1]	Bulk partition coefficients Di[2]	Enriched melt components Macquarie[3]	Rurutu[4]	Mantle restites[5] Degree of 1st stage melting 5%	10%	2nd stage melts[6] Amount of Rurutu-type component in source 0%	0.05%	1%
**Rb**	0.635	0.00010	59.5	19	1.2E-223	0.0E+00	0.0E+00	0.109	2.17
**Ba**	6.989	0.00012	584	264	1.7E-185	0.0E+00	0.0E+00	1.51	30.2
**Th**	0.085	0.001	7.04	4.88	4.7E-24	1.7E-47	1.4E-49	0.028	0.558
**U**	0.021	0.0011	1.94	1.3	1.2E-22	5.9E-44	9.7E-46	0.007	0.149
**Nb**	0.713	0.0034	91.4	62.3	2.1E-07	2.8E-14	3.8E-14	0.356	7.1
**Ta**	0.041	0.0034	5.08	3.65	1.2E-08	1.6E-15	2.2E-15	0.021	0.418
**La**	0.687	0.01	46.6	46.5	4.3E-03	2.0E-05	1.1E-04	0.266	5.32
**Ce**	1.775	0.022	86.3	91.9	0.182	0.016	0.134	0.650	10.5
**Pb**	0.185	0.025 (0.014)	2.32	2.48	0.025	0.003	0.025	0.039	0.301
**Pr**	0.276	0.027	10.1	11.5	0.043	0.006	0.053	0.116	1.32
**Nd**	1.354	0.031	33.0	52.8	0.272	0.050	0.433	0.717	6.12
**Sr**	21.1	0.031 (0.025)	703	820	4.25	0.783	6.74	11.2	95.1
**Zr**	11.2	0.033	163	238	2.49	0.511	4.41	5.67	29.7
**Hf**	0.309	0.035	3.66	5.85	0.075	0.017	0.146	0.177	0.760
**Sm**	0.444	0.045	5.93	11.3	0.149	0.047	0.399	0.454	1.502
**Eu**	0.168	0.050	2.01	3.74	0.063	0.023	0.187	0.204	0.537
**Ti**	1300	0.050 (0.058)	11913	21300	491	176	1444	1543	3437
**Gd**	0.596	0.056	5.12	11.2	0.251	0.101	0.804	0.853	1.80
**Tb**	0.108	0.068	0.80	1.62	0.053	0.025	0.189	0.195	0.319
**Dy**	0.737	0.079	4.39	9.26	0.405	0.216	1.49	1.52	2.17
**Ho**	0.164	0.084	0.90	1.72	0.094	0.052	0.347	0.353	0.468
**Y**	4.55	0.088	22.6	50.8	2.67	1.53	9.93	10.1	13.4
**Er**	0.48	0.097	2.35	4.32	0.298	0.180	1.10	1.12	1.38
**Yb**	0.493	0.115	2.13	2.92	0.332	0.219	1.20	1.21	1.36
**Lu**	0.074	0.12	0.31	0.44	0.051	0.034	0.181	0.182	0.204

[1] Primitive mantle composition from Sun & McDonough (1989)

[2] Bulk partition coefficients during partial mantle melting after Workmann and Hart (2005) except for Pb, Sr and Ti, which were modified slightly to provide less spiked spectra during fractional melting.

Original Di values for these elements from Workman and Hart (2005) are given in parentheses.

[3] Sample #479632 from Kamenetsky et al. (2000)

[4] Sample #sRRT-B-303 from Willbold & Stracke (2006)

[5] Residual mantle composition after 5% and 10% of fractional melting of PM

[6] Pooled fractional melts produced by 8.7 % melting of 10.6% restite after PM melting comprising to 0 wt%, 0.05 wt% and 1 wt% of enriched in incompatible trace elements Rurutu-type melt component.

Supplementary Table 5: Estimated isotopic composition of the UDM and enriched melt end members

	SO193DR26-1[2,3]	SO193DR26-3[2]	SO193DR26-10[2]	SO225DR12-2	SO225DR12-3	SO225DR24-1	SO225DR24-10	Estimated melt end member compositions	
								UDM	enriched (Rurutu-type)
$^{206}Pb/^{204}Pb(t)$[1]	19.714	19.721	19.711	19.699	19.779	18.709	18.732	17.980	19.871
2σ								0.156	0.156
$^{207}Pb/^{204}Pb(t)$[1]	15.630	15.650	15.647	15.645	15.651	15.547	15.553	15.480	15.660
2σ								0.027	0.027
$^{208}Pb/^{204}Pb(t)$[1]	39.058	39.087	39.037	39.056	39.116	38.438	38.557	38.070	39.166
2σ								0.109	0.109
$^{87}Sr/^{86}Sr(t)$[1]	0.702870	0.702885	0.702872	0.702818	0.702820	0.703533	0.703507	0.703733	0.702797
2σ								0.000072	0.000072
$^{143}Nd/^{144}Nd(t)$[1]	0.512788	0.512790	0.512796	0.512804	0.512788	0.512830	0.512832	0.512851	0.512786
2σ								0.000009	0.000009
εNd(t)[1]	5.94	5.98	6.09	6.26	5.94	6.76	6.79	-	-
2σ									

Notes:

[1] t=120Ma

[2] isotope data from Timm et al., 2011 (except for Sr isotope ratio in sample SO193DR26-1)

[3] newly generated Sr isotope data

Note: 2sigma errors for the estimated isotopic composition of the melt end members were calculated as the sum of squares of the deviation of each sample/data point from the regression line.

Supplementary Table 6a: EMP data on reference samples

Comment	VGA99 (mean) n=8	1s	VGA99_ ref[1]	VG-2 (mean) n=8	1s	VG2_ref[1]
**SiO_2**	50.48	0.45	50.94	50.41	0.48	50.81
**TiO_2**	4.05	0.05	4.06	1.88	0.03	1.85
**Al_2O_3**	12.32	0.15	12.49	13.82	0.21	14.06
**FeO_t**	13.17	0.42	13.30	11.79	0.24	11.84
**MnO**	0.17	0.04	0.15	0.24	0.05	0.22
**MgO**	5.09	0.07	5.08	7.00	0.06	6.71
**CaO**	9.41	0.05	9.30	11.34	0.06	11.12
**Na_2O**	2.75	0.11	2.66	2.73	0.12	2.62
**K_2O**	0.83	0.02	0.82	0.19	0.01	0.19
**P_2O_5**	0.42	0.03	0.38	0.21	0.04	0.20
**SO_3**	0.03	0.02	-	0.34	0.05	0.35[2]
**Cl**	0.03	0.01	-	0.03	0.01	0.03[2]
**F**	0.05	0.06	-	0.01	0.02	0.03[2]
**Total**	98.82			99.99		

Notes:

[1] reference values from Jarosewich et al. (1980)

[2] reference values from SIMS analyses from Sobolev et al. (2016)

Supplementary Table 6b: Standard reference material analyses for XRF

	JGB-1	JGB-1	JGB-1	average	stdev	GSJ JGb-1[1]	JB-3	JB-3	JB-3	average	stdev	GSJ JB-3[1]	JB-2	JB-2	JB-2	average	stdev	GSJ JB-2[1]
**SiO_2**	43.97	43.87	43.8	43.88	0.09	43.66	50.63	50.51	50.68	50.61	0.09	50.96	54.09	53.76	53.78	53.88	0.19	53.25
**Al_2O_3**	17.41	17.54	17.65	17.53	0.12	17.49	16.89	16.98	17.22	17.03	0.17	17.2	14.68	14.63	14.78	14.70	0.08	14.64
**Fe_2O_3**	15.07	15.38	15.34	15.26	0.17	15.06	11.64	11.79	11.83	11.75	0.10	11.82	14.23	14.43	14.43	14.36	0.12	14.25
**MnO**	0.19	0.19	0.19	0.19	0.00	0.189	0.17	0.18	0.18	0.18	0.01	0.18	0.22	0.22	0.22	0.22	0.00	0.22
**MgO**	7.72	7.78	7.93	7.81	0.11	7.85	5.05	5.03	5.13	5.07	0.05	5.19	4.57	4.58	4.64	4.60	0.04	4.62
**CaO**	11.72	11.86	11.91	11.83	0.10	11.90	9.60	9.69	9.71	9.67	0.06	9.79	9.78	9.83	9.87	9.83	0.05	9.82
**Na_2O**	1.30	1.25	1.28	1.28	0.03	1.20	2.77	2.71	2.81	2.76	0.05	2.73	2.11	2.05	2.10	2.09	0.03	2.04
**K_2O**	0.23	0.23	0.23	0.23	0.00	0.24	0.75	0.75	0.76	0.75	0.01	0.78	0.41	0.41	0.42	0.41	0.01	0.42
**TiO_2**	1.61	1.62	1.62	1.62	0.01	1.60	1.43	1.43	1.43	1.43	0.00	1.44	1.19	1.19	1.18	1.19	0.01	1.19
**P_2O_5**	0.05	0.05	0.06	0.05	0.01	0.06	0.29	0.28	0.29	0.29	0.01	0.29	0.09	0.10	0.10	0.10	0.01	0.10
**SO_3**	0.20	0.27	0.28	0.25	0.04		0.01	0.07	0.09	0.06	0.04		0.01	0.07	0.08	0.05	0.04	
**L.O.I**	0.85	0.85	0.85	0.85	0.00		0.00	0.00	0.00	0.00	0.00		0.00	0.00	0.00	0.00	0.00	
**SUM**	100.32	100.89	101.14				99.23	99.42	100.13				101.38	101.27	101.6			

	JA-3	JA-3	JA-3	average	stdev	GSJ JA-3[1]	JG-3	JG-3	JG-3	average	stdev	GSJ JG-3[1]
**SiO_2**	61.71	61.24	61.12	61.36	0.31	62.27	67.19	67.18	67.04	67.14	0.08	67.29
**Al_2O_3**	15.2	15.18	15.28	15.22	0.05	15.56	15.25	15.25	15.50	15.33	0.14	15.48
**Fe_2O_3**	6.33	6.43	6.44	6.40	0.06	6.60	3.66	3.72	3.71	3.70	0.03	3.69
**MnO**	0.10	0.10	0.10	0.10	0.00	0.10	0.07	0.07	0.07	0.07	0.00	0.07
**MgO**	3.58	3.58	3.65	3.60	0.04	3.72	1.76	1.79	1.82	1.79	0.03	1.79
**CaO**	6.11	6.12	6.17	6.13	0.03	6.24	3.74	3.76	3.76	3.75	0.01	3.69
**Na_2O**	3.15	3.09	3.16	3.13	0.04	3.19	4.15	4.04	4.16	4.12	0.07	3.96
**K_2O**	1.37	1.36	1.37	1.37	0.01	1.41	2.62	2.62	2.63	2.62	0.01	2.64
**TiO_2**	0.68	0.68	0.68	0.68	0.00	0.70	0.48	0.48	0.48	0.48	0.00	0.48
**P_2O_5**	0.11	0.11	0.11	0.11	0.00	0.12	0.12	0.13	0.13	0.13	0.01	0.12
**SO_3**	0.04	0.10	0.11	0.08	0.04		0.01	0.08	0.10	0.06	0.05	
**L.O.I**	0.34	0.34	0.34	0.34	0.00		0.93	0.93	0.93	0.93	0.00	
**SUM**	98.72	98.33	98.53				99.98	100.05	100.33			

Notes:
[1] reference data from Itoh et al., 1995, Geostandards Newsletter

Supplementary Table 6c: LA-ICP-MS data on reference samples

Conc. in ppm	Mass number	KL2-G GEOREM n=36	1s	G128-G Mean n=6	1s	GEOREM	BCR-2G Mean n=8	1s	GEOREM
**Li**	7	5.10	0.19	10.2	0.68	10.4	7.90	0.23	9.00
**Ca (ref)**	43	10.9	-			6.24			7.06
**Sc**	45	31.8	0.66	32.7	1.00	32.1	33.8	1.32	33.0
**Ti**	47	15360	404	1744	38.2	1728	13799	387	13620
**V**	51	309	7.37	192.16	7.83	189	413	15.4	425
**Cr**	52	294	7.85	2424	106	2272	21.0	4.48	17.0
**Co**	59	41.2	1.04	95.8	4.11	92.4	35.1	0.94	38.0
**Ni**	60	112	3.38	1232	24.0	1074	11.5	0.76	13.0
**Cu**	63	87.9	3.16	69.3	1.40	63.8	16.5	2.00	21.0
**Ga**	71	20.0	0.64	9.66	0.13	8.67	21.2	0.85	23.0
**As**	75	0.15	0.06	0.06	0.03	0.10	1.08	0.24	-
**Rb**	85	8.70	0.26	0.43	0.01	0.41	46.8	1.69	47.0
**Sr**	88	356	11.8	30.6	0.48	30.0	337	8.87	342
**Y**	89	25.4	1.18	12.5	0.41	11.8	34.9	1.83	35.0
**Zr**	90	152	7.28	10.2	0.39	10.0	176	12.7	184
**Nb**	93	15.0	0.43	0.11	0.02	0.10	12.6	0.28	12.5
**Mo**	98	3.60	0.15	0.86	0.09	0.71	275	29.7	270
**Sn**	120	1.54	0.06	0.20	0.01	0.22	2.22	0.24	2.60
**Sb**	121	0.14	0.01	0.03	0.03	0.01	0.32	0.03	0.35
**Cs**	133	0.11	0.00	0.23	0.01	0.24	1.10	0.04	1.16
**Ba**	138	123	4.90	1.21	0.17	1.06	621	53.9	683
**La**	139	13.1	0.49	0.13	0.03	0.12	24.5	0.74	24.7
**Ce**	140	32.4	1.05	0.48	0.06	0.45	51.7	1.39	53.3
**Pr**	141	4.60	0.18	0.10	0.01	0.10	6.65	0.16	6.70
**Nd**	146	21.6	0.91	0.79	0.04	0.78	28.3	0.75	28.9
**Sm**	147	5.54	0.27	0.54	0.02	0.53	6.44	0.19	6.59
**Eu**	151	1.92	0.08	0.27	0.01	0.26	1.92	0.03	1.97
**Gd**	157	5.92	0.31	1.21	0.07	1.17	6.68	0.33	6.71
**Tb**	159	0.89	0.05	0.26	0.01	0.25	1.04	0.06	1.02
**Dy**	163	5.22	0.28	1.97	0.11	1.98	6.41	0.32	6.44
**Ho**	165	0.96	0.05	0.45	0.02	0.44	1.27	0.07	1.27
**Er**	166	2.54	0.16	1.40	0.06	1.40	3.66	0.22	3.70
**Tm**	169	0.33	0.02	0.21	0.01	0.20	0.52	0.03	0.51
**Yb**	172	2.10	0.13	1.41	0.06	1.41	3.52	0.16	3.39
**Lu**	175	0.28	0.02	0.21	0.01	0.21	0.52	0.03	0.50
**Hf**	178	3.93	0.24	0.35	0.02	0.35	4.99	0.25	4.84
**Ta**	181	0.96	0.05	0.02	0.00	0.02	0.82	0.02	0.78
**W**	184	0.33	0.12	13.6	0.43	15.5	0.42	0.01	0.50
**Pb**	208	2.07	0.10	0.34	0.01	0.35	11.6	0.73	11.0
**Th**	232	1.02	0.06	0.01	0.01	0.01	6.13	0.17	5.90
**U**	238	0.55	0.03	0.01	0.00	0.01	1.71	0.12	1.69

Notes:
All data were calibrated against reference glass KL-2G.
Glasses G128-G and BCR-2G were analysed as unknown.
Reference data (GEOREM column) are preferred values from GEOREM data base of geologic reference materials (http://georem.mpch-mainz.gwdg.de/sample_query_pref.asp)
1s - one standard deviation of the mean values

Supplementary references

1 Stracke, A., Hofmann, A. W. & Hart, S. R. Fozo, Himu, and the Rest of the Mantle Zoo. *Geochemistry Geophysics Geosystems* **6**, 1-20, doi:10.1029/2004gc000824 (2005).

2 Willbold, M. & Stracke, A. Trace element composition of mantle end-members: Implications for recycling of oceanic and upper and lower continental crust. *Geochemistry Geophysics Geosystems* **7**, doi:10.1029/2005gc001005 (2006).

3 Tejada, M. L. G., Mahoney, J. J., Neal, C. R., Duncan, R. A. & Petterson, M. G. Basement Geochemistry and Geochronology of Central Malaita, Solomon Islands, with Implications for the Origin and Evolution of the Ontong Java Plateau. *Journal of Petrology* **43** (2002).

4 Tejada, M. L. G. *et al.* Pin-pricking the elephant: evidence on the origin of the Ontong Java Plateau from Pb-Sr-Hf-Nd isotopic characteristics of ODP Leg 192 basalts. *Fitton J. G., Mahoney, J. J., Wallace, P. J., Saunders, A. D. (eds), Origin and Evolution of the Ontong Java Plateau: Geological Society, London, Special Publications, 229, pp. 133-150.* (2004).

5 Meyzen, C. M. *et al.* Isotopic portrayal of the Earth's upper mantle flow field. *Nature* **447**, 1069-1074, doi:10.1038/nature05920 (2007).

6 Timm, C. *et al.* Age and geochemistry of the oceanic Manihiki Plateau, SW Pacific: New evidence for a plume origin. *Earth and Planetary Science Letters* **304**, 135-146, doi:10.1016/J.Epsl.2011.01.025 (2011).

7 Workman, R. K. & Hart, S. R. Major and trace element composition of the depleted MORB mantle (DMM). *Earth and Planetary Science Letters* **231**, 53-72, doi:10.1016/j.epsl.2004.12.005 (2005).

8 Stracke, A., Bizimis, M. & Salters, V. J. M. Recycling oceanic crust: Quantitative constraints. *Geochemistry Geophysics Geosystems* **4**, 1-33, doi:10.1029/2001gc000223 (2003).

9 Langmuir, C. H., Vocke, R. V., Hanson, G. N. & Hart, S. R. A general mixing equation with applications to Icelandic basalts. *Earth and Planetary Science Letters* **37**, 380-392 (1978).

10 Kamenetsky, V. S. *et al.* Enriched end-member of primitive MORB melts: petrology and geochemistry of glasses from Macquarie Island (SW Pacific). *Journal of Petrology* **41**, 411-430, doi:10.1093/Petrology/41.3.411 (2000).

11 McDonough, W. F. & Sun, S. S. The Composition of the Earth. *Chemical Geology* **120**, 223-253, doi:10.1016/0009-2541(94)00140-4 (1995).

12 Kerrich, R. & Xie, Q. L. Compositional recycling structure of an Archean super-plume: Nb-Th-U-LREE systematics of Archean komatiites and basalts revisited. *Contributions to Mineralogy and Petrology* **142**, 476-484 (2002).

13 O'Neil, J., Francis, D. & Carlson, R. W. Implications of the Nuvvuagittuq Greenstone Belt for the Formation of Earth's Early Crust. *Journal of Petrology* **52**, 985-1009, doi:10.1093/petrology/egr014 (2011).

14 Polat, A., Hofmann, A. W. & Rosing, M. T. Boninite-like volcanic rocks in the 3.7-3.8 Ga Isua greenstone belt, West Greenland: geochemical evidence for intra-oceanic subduction zone processes in the early Earth. *Chemical Geology* **184**, 231-254, doi:10.1016/S0009-2541(01)00363-1 (2002).
